# Evaluating user experiences with an AI chatbot for health-related social needs: A cross-sectional mixed methods study

**DOI:** 10.1371/journal.pdig.0001789

**Published:** 2026-10-30

**Authors:** Emre Sezgin, Daniel I. Jackson, Syed-Amad Hussain, A. Baki Kocaballi, Carson Richardson, Micah Skeens, Eric Fosler-Lussier, Aldenise P. Ewing, Macarius Donneyong, Ahna Pai

**Affiliations:** 1 Center for Biobehavioral Health, The Abigail Wexner Research Institute at Nationwide Children’s Hospital, Columbus, Ohio, United States of America; 2 Department of Pediatrics, The Ohio State University, College of Medicine, Columbus, Ohio, United States of America; 3 Department of Computer Science and Engineering, The Ohio State University, College of Engineering, Columbus, Ohio, United States of America; 4 School of Computer Science, University of Technology Sydney, Faculty of Engineering and Information Technology, Sydney, Australia; 5 Center for Biostatistics, The Ohio State University, Wexner Medical Center, Columbus, Ohio, United States of America; 6 Division of Epidemiology, The Ohio State University, College of Public Health, Columbus, Ohio, United States of America; 7 Division of Outcomes and Translational Sciences, The Ohio State University, College of Pharmacy, Columbus, Ohio, United States of America; The Chinese University of Hong Kong, HONG KONG

## Abstract

Unmet health-related social needs (hrsns) significantly impact health. while screening tools exist, linking individuals to resources remains difficult. chatbots offer a potential solution for personalized, real-time navigation, but their usability and trustworthiness, particularly among high-need populations, need further study. we conducted a mixed-methods study with 128 caregivers to evaluate an ai chatbot, daphne, designed to identify hrsns and recommend resources. data on usability, task load, satisfaction, and trust were collected, along with qualitative feedback and conversation transcripts. regression and pairwise analyses explored associations between demographics, hrsns, and user experience. participants were mostly female (68%), 30–49 (71%), white (44%) or black/african american (36%), relied on medicaid/medicare (83%), and cared for a child with special healthcare needs (78%). they reported high usability (sus = 84.7), low task load (nasa-tlx = 6.8), high satisfaction (nps = 8.0), and high trust (mean = 4.1). nearly all (98%) reported unmet hrsns, including food insecurity (76%) and financial limitations (75%). chatbot sessions averaged 3 minutes/20 turns. daphne achieved 99% intent accuracy in 1,523 turns, focusing on financial, housing, and food needs. usability and trust were consistent across demographics, though higher education and lower income groups showed slight usability decreases. certain hrsns (transportation, utility disruptions) correlated with higher trust and satisfaction, suggesting particular value for users facing structural barriers. daphne demonstrated strong usability and trust across diverse groups. future designs should focus on longitudinal impact and effectiveness for improved accessibility and practical implementation. our study supports digital social care practices and provides empirical evidence that a chatbot can engage vulnerable populations to support unmet hrsns.

## Author summary

Many families caring for children struggle with everyday challenges such as finding enough food, paying utility bills, securing housing, or getting reliable transportation. These social needs strongly affect health, yet they are usually screened for only during clinic visits, often once a year, leaving families without support when problems arise between visits. Even when needs are identified, caregivers frequently have difficulty finding and accessing available assistance programs. In this study, we tested whether a conversational AI chatbot, called DAPHNE, could help caregivers identify their social needs and connect to relevant resources outside the clinic. Caregivers interacted with the chatbot using realistic scenarios based on common, real-world challenges. They could type freely or use on-screen buttons, similar to everyday messaging apps. We found that caregivers across diverse backgrounds generally found the chatbot easy to use, trustworthy, and efficient. Those facing challenges related to transportation or utilities reported especially high trust and satisfaction. Interactions were brief but effective, suggesting the chatbot fit well into caregivers’ daily lives. Our findings show that a purpose-built healthcare chatbot can support families by improving access to social resources beyond traditional clinic-based screening.

## Introduction

Health-related social needs (HRSNs), such as housing stability, transportation, food security, access to healthcare, and financial stability, account for over half of modifiable health outcomes and are closely associated with elevated morbidity, increased healthcare utilization, and premature mortality [1–3]. These needs cluster along entrenched lines of income, race, and geography, amplifying inequities in health outcomes [4,5]. Children are particularly vulnerable: persistent material deprivation impairs physical, emotional, and cognitive development and predicts poorer adult health outcomes [4–7]. Routine HRSN screening is recommended across pediatric and adult healthcare settings, prompting widespread adoption of brief screeners in primary care, emergency departments, and inpatient units [7]. However, screening alone is insufficient; linking families to community resources remains a critical challenge [8,9].

In many clinics, a positive screening yields only brief provider guidance and a printed “resource sheet,” leaving patients and caregivers to navigate a fragmented constellation of supports. Staffing constraints, language discordance, and shifting eligibility requirements often derail this passive model - fewer than half of referred patients ultimately receive services [10,11]. While electronic health record-embedded screeners and patient portals enhance data capture, their effectiveness is limited without integration into a dynamic referral system. Their reliance on static directories and manual matching heightens the risk of outdated information and missed connection [12–14]. Conversational agents (chatbots) can bridge the gap in understanding needs and navigating resources by coupling natural-language processing with adaptive dialogue to deliver tailored guidance at scale [15,16]. Across the literature of chatbot use in healthcare, systematic reviews and pilot studies focusing on health-information seeking, preventive screening, medication adherence, and chronic-disease self-management consistently report high perceived usability and promising clinical impact [17–21].

Our prior iterative design and evaluation study of a semi-functional and rule-based HRSN chatbot, DAPHNE (Dialog-based Assistant Platform for Healthcare and Needs Ecosystem), demonstrated perceived utility for resource linkage but also highlighted concerns regarding accuracy, resource sharing, and user trust among adult caregivers [18]. The current study builds upon this earlier work with an improved functionality chatbot (LLM-based application). This study aimed to evaluate the user experience associated with the DAPHNE chatbot among caregivers with unmet HRSNs. We sought to understand how users engage with an AI-powered social care assistant and to identify demographic or contextual factors associated with varied user experiences. The findings of this study will inform future design enhancements and implementation strategies for equitably scaling digital social care interventions across diverse clinical and community settings.

## Methods

### Ethics statement

This study received approval to conduct human-subjects research (STUDY00004369) from The Internal Review Board (FWA000002860) at Nationwide Children’s Hospital. Research staff obtained written informed consent electronically completed surveys to determine eligibility.

### Study design

We conducted a mixed-methods study with a convergent parallel design to evaluate DAPHNE chatbot [22]. The primary outcomes were user experience with measures of usability, satisfaction, task load, and trust, while examining how sociodemographic factors and identified HRSNs moderate these outcomes. The reporting of this mixed-methods observational study was guided by the **S**trengthening **T**he **R**eporting of **OB**servational studies in **E**pidemiology (STROBE) [23] checklist, ensuring transparency and completeness in the presentation of methods and results (See S1 Checklist). Fig 1 outlines the overall study design and procedures.

**Fig 1:**
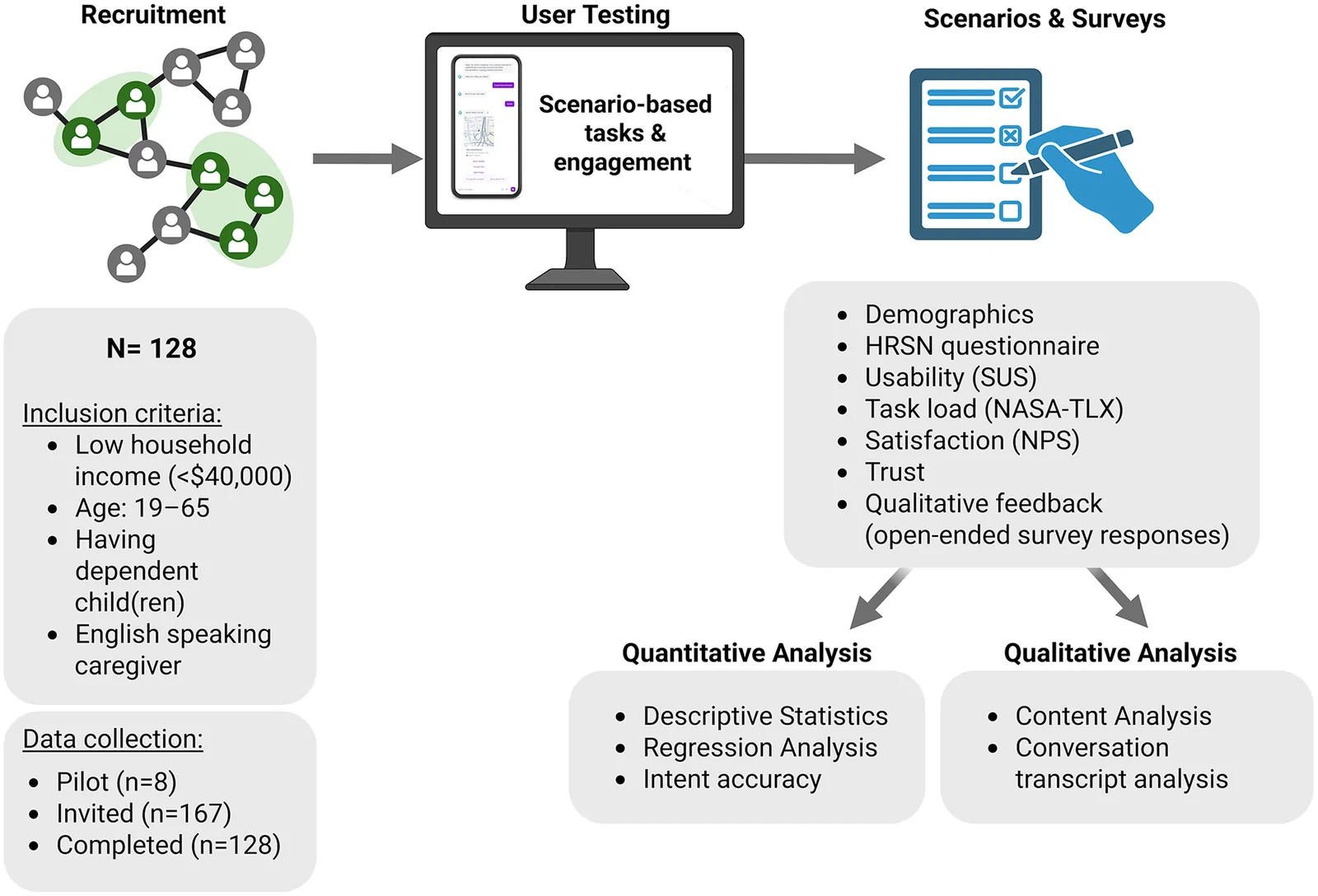
Study design, procedure and data collection.

### DAPHNE chatbot

The DAPHNE chatbot was developed to identify unmet HRSNs and provide personalized, location-based community resource recommendations via preferred natural language. The chatbot employed a semi-structured conversational interface that supported both free-text and button-based inputs, allowing flexible user interaction and navigating community resources. After completing each session, participants were prompted to rate their experience and provide feedback, including both the quality of resource information and the conversational flow.

To design and prototype the dialog system, Amazon Web Services (AWS) was initially used to test conversation flows and explore integration of early large language model (LLM) capabilities, including intent recognition and conversational responses. Once core functions were finalized on AWS, the chatbot was adapted into a web app (accessible via desktop or mobile device web browser) using Voiceflow platform that supports secure, browser-based deployment and dialog management, including integration with third-party APIs and LLMs for natural language tasks (see S11 Appendix). Fig 2 provides an outline of DAPHNE chatbot and its ecosystem.

**Fig 2:**
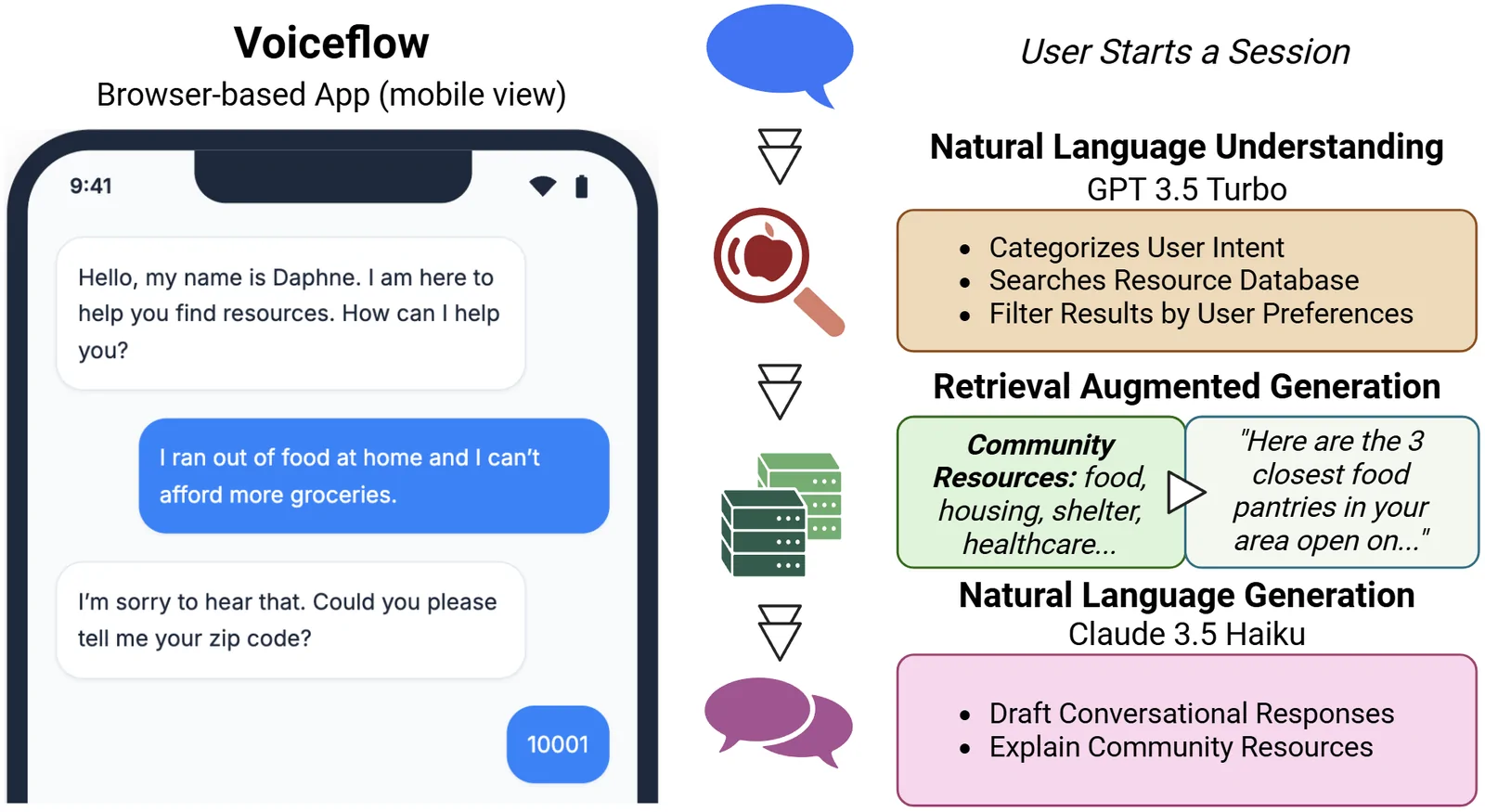
Overview of the DAPHNE chatbot. This system uses Natural Language Understanding and Application Programming Interface for its functionality and to augment existing HRSN support.

To accommodate variability in user interaction styles (such as button selection versus typed input) the chatbot used a hybrid dialogue architecture that combined rule-based logic for structured flows (e.g., asking for zip code, presenting resource categories) with Natural Language Understanding (NLU) for more open-ended queries. For example, if a user typed, “I need help paying rent,” the NLU module mapped this intent to the “housing support” domain and triggered the corresponding response logic. NLU and Natural Language Generation (NLG) were powered by large language models (OpenAI’s GPT-3.5 Turbo and Anthropic’s Claude 3.5 Haiku) [24,25], which helped interpret ambiguous input, clarify misunderstood queries, and provide context-aware language in follow-up prompts. Our dual model approach was driven by our earlier experiments (unpublished) which indicated GPT-3.5 Turbo performs better in intent recognition while Claude 3.5 Haiku performs better in response generation. In cases where the chatbot could not confidently categorize a user message- for example, if the input did not match any predefined intent or the user entered a vague or unrelated phrase- this was flagged as a non-categorized entry, triggering a fallback script. The fallback response informed users that the chatbot could not understand their request and prompted them to try again or use button options.

Recommendations were generated based on data retrieved from a live social-care API (Findhelp.org), which contained up-to-date community resource listings. The chatbot only used information returned from the API and did not generate recommendations independently, ensuring response accuracy and grounding in real-world availability. Chatbot messages were informed by pre-written/scripted responses. While the API supplied information about resources, LLMs conveyed scripts with conversational language. Some user requests, such as highly specific eligibility questions, were occasionally not available through the API, and in such cases, users were informed accordingly.

This structured query-and-response design follows the Retrieval-Augmented Generation (RAG) framework provided by the platform, which combines real-time information retrieval with natural language output [26,27]. The chatbot generates an API template during the conversation. After the chatbot narrows a user’s need to a category of resources, it sends the generated API call, which returns with information from their social care database. When referring to retrieved resources in conversation, the chatbot prepares interactive resource cards to consolidate information related to each service. RAG is an architectural approach in which model outputs are conditioned on externally retrieved information rather than relying solely on a model’s pretrained knowledge. In DAPHNE, RAG was implemented in a constrained manner to ensure grounding and accuracy in resource recommendations. Specifically, user inputs were first processed to identify intent and relevant entities (e.g., need type, zip code), which were then used to retrieve up-to-date community resource data from the API.

### Recruitment and procedure

We conducted an a priori power analysis using G*Power 3.1 [28] to determine the minimum sample size needed to detect the study’s primary outcomes. A fixed-model multiple linear regression analysis indicated that 129 participants would be required for each User Experience outcome, assuming a medium effect size (f^2^ = 0.15), α = 0.05, target power = 0.95, and 4 predictors. Participants were recruited through an online research platform (Prolific.com) following a purposeful sampling approach. Recruitment and study participation occurred between January 1st and March 30th, 2025. Inclusion criteria required participants to be caregivers aged 19 years or older, residing in the U. S., caring for at least one dependent child, and reporting an annual household < $40,000 (<200% of the 2025 United States Federal Poverty Level). Participants were excluded from the study if they declined consent, opted out at any step of the study, or failed attention-check tasks embedded within chatbot interactions. Informed consent was obtained electronically, and participants completed study participation via a HIPAA (Health Insurance Portability and Accountability Act) compliant, online survey platform (Research Electronic Data Capture [REDCap]; a web-based software tool for collecting and organizing research data) [29]. Participants were compensated for their time ($12). Recruitment ended after exhausting funding for compensation.

Participants received their instructions and completed quantitative and qualitative measures on REDCap before and after engaging with the chatbot. Engagement with the chatbot was guided through four scenarios. Prior to the data collection, a pilot group of participants (N = 8) were recruited to test for understandability and clarity of survey questions, scenarios and chatbot conversations. In response to their feedback, we revised the study instructions in the survey, added clarifications to HRSN-related questions, and refined scenario content to improve understandability. The data from pilot participants were not included in the analysis.

### Scenarios

The study team designed four scenarios, informed by our previous investigation [18], to simulate real-world cases and to demonstrate the chatbot’s capabilities. Scenarios were used to standardize the content that the participants engaged with (details in S1 Appendix). The scenario topics were selected purposefully to reflect high-priority HRSNs - food insecurity, healthcare access, and housing instability - that align with Center for Medicare and Medicaid Services (CMS) screening requirements and commonly targeted domains in Medicaid and health system initiatives [30].

In addition, each scenario tested a distinct chatbot function (e.g., location-based search, cost inquiry, service coverage), allowing structured evaluation of key features. The final open-ended task enabled observation of natural user behavior, offering a balanced assessment of performance and real-world engagement. Specifically, the first scenario required participants to identify food and nutritional assistance programs and obtain information about the operational hours of these programs based on their residential zip codes. In the second scenario, participants explored healthcare-related resources in a new zip code and requested information regarding the new program’s cost of service. The third scenario involved participants seeking housing support resources in a nearby city, focusing specifically on determining service coverage and obtaining relevant contact information. Following the completion of these structured scenarios, the last scenario instructed participants to freely engage and explore the chatbot’s capabilities in line with their personal interests or social needs.

### Data collection

Quantitative data collection included demographic characteristics, HRSNs, and user experience. The HRSNs assessed in the survey comprised food insecurity, housing instability, transportation barriers, financial strain, social isolation, and health literacy deficits. Items for measuring HRSNs were adapted from established screening instruments utilized by the U.S. Census Bureau [31], advocacy organizations [32–34], healthcare systems [35,36], research institutes [37–41], and community health centers. User experience was assessed using four standardized instruments: the National Aeronautics Space Administration Task Load Index (NASA-TLX; 6-item measure for perceived cognitive and workload demands during task completion) [42], System Usability Scale (SUS; 10-item scale that evaluates perceived usability of interactive systems) [43], Net Promoter Score (NPS; single item question for satisfaction and willingness to recommend the system) [44], and a Trust scale (adapted from Jian et al. [45]; single item question assessing perceived confidence in the chatbot’s ability to provide accurate information).

Qualitative data was collected via open-ended responses within REDCap. Specifically, participants provided narrative responses to describe a positive experience, a negative experience, and suggested improvements for the chatbot. In addition, transcripts of participant- chatbot interactions were recorded (2-way verbatim dialogue texts including dialog turn labels, metadata, timestamps).

### Data analysis

We reported descriptives of demographics, HRSN and user experience responses. In addition, we conducted qualitative analysis of open-ended survey responses, conversational analysis using transcripts of chatbot-user engagement, calculated chatbot intent accuracy and finally exploratory analysis to investigate the association between demographics, HRSN vs. user experience outcomes via univariable regression models.

### Qualitative analysis

Qualitative data were two-fold: (1) Free-text feedback over the survey and (2) chatbot conversation transcripts. Free-text feedback was analyzed for sentiment (i.e., positive, negative, and neutral) and intensity (i.e., high, low) to highlight interactions with the chatbot. Sentiment was rated by the study team (AH, DIJ) on a Likert scale from 1 (Negative) to 5 (Positive) indicating the level of satisfaction in the user’s language. Intensity similarly coded by the study team on a Likert scale from 1 (Low) to 5 (High) indicating the level of emotional content in user’s language (see S2 Appendix). Furthermore, we conducted a thematic analysis, developing an initial codebook through an inductive review of 10% of the transcripts (DJ, AH). Codes were refined iteratively after each transcript was reviewed. After the final transcript, codes were reviewed once more to define our themes. Transcript data was processed through Python [46], and coding of the dataset was done through Microsoft Excel. Interrater reliability was assessed using Cohen’s Kappa (a statistical measure for assessing agreement between two raters beyond what would occur by chance) [47]. Any disputes were resolved by a senior team member (ES).

### Conversational analysis

Chatbot conversation transcripts were coded and manually analyzed by research team members (DIJ, AH) using a structured rubric to categorize patterns in resource-seeking behaviors for each free-form conversational turn. The defined rubric notes when users mention (1) a specific resource, (2) the purpose for the resource, (3) their personal background, (4) multiple needs, and (5) thoughts on finding a resource (see S3 Appendix). Each behavior was operationalized as a binary variable, with transcripts coded as 1 when the behavior was present and 0 when it was absent. A *specific resource* included a title or descriptor to explain their need (for example, “allergen-free food”). A *purpose for the resource* included mentions on who or why the resource is necessary (“for my friend”). The *personal background* referred to contextual details about their circumstances (“I just moved”). *Multiple needs as a resource-seeking* behavior including at least two distinct needs that would require different types of assistance (such as across multiple HRSNs). Finally, we had the category for participants expressing *their thoughts on finding a resource* with implicit or explicit intent of exploring community resources.

### Intent accuracy

To validate the chatbot’s NLU accuracy (ability to recognize HRSNs from the user’s free-form entry), the study team calculated user “intents” as a percentage of accuracy. For each message that requires a fallback mechanism from the chatbot to correctly identify the topic of interest, the intent is approximated out of the possible services available in the API. We automatically tagged each occurrence in the chatbot’s infrastructure while exporting transcript data.

### Regression analysis

Chatbot usability, satisfaction, and task load were modeled via linear regression, whereas chatbot trustworthiness was modeled using an ordinal logistic regression. More specifically, we performed category-wise regressions where the coefficients of each variable in the category are compared against the category’s reference variable (RV).

For each demographic category, regression coefficients were interpreted relative to a reference variable (RV). Demographic variables included both binary (e.g., Insurance: Yes/No) and categorical predictors with multiple response levels (e.g., Marital Status). For multi-level categorical predictors, we highlight response options with “statistically significant” coefficients (i.e., 95% confidence intervals that do not cross the null value defined by the RV). Where appropriate, we also present binarized contrasts (e.g., Married vs. all other marital statuses). Furthermore, for trust factor, we presented predicted mean trust level to directly correlate level of trust to each demographic and HRSN group in **Tables A-G in** S10 Appendix.

We additionally conducted a pairwise analysis between variables within a given category (e.g., social isolation) to quantify suggestive trends between user demographics and HRSN against our user experience metrics. Pairwise results were noted in terms of differences between variable means (Beta difference for Usability, Task Load, and Satisfaction) and are presented in **Tables A-U in** S9 Appendix. Suggestive trends were presented either as monotonically scaling, especially for ordinal variables (e.g., education or income), or general shifts. All statistical analyses were conducted using statistical software, R (v4.5.0).

### Mixed-methods interpretation

Following our convergent parallel design, we interpreted quantitative survey results with qualitative feedback and transcript-based conversational analytics. Specifically, we compared findings across data sources to identify areas of convergence (where qualitative comments aligned with survey ratings) and divergence (where qualitative findings explained limitations not captured by summary metrics), and we used these comparisons to synthesize results into discussion and implementation implications.

## Results

We recruited 167 participants to the study, and 128 participants (76.6%) completed all activities and were included in the analysis.

### Demographics

Participants were geographically distributed across diverse urban and rural settings throughout the U.S. (Table 1). The sample (N = 128) was predominantly White (44%) and Black/African American (36%), with 27% identifying as Hispanic or Latino. Most participants identified as female (68%), were between 30 and 49 years old (71%), and primarily spoke only English (78%). Although 22% were bilingual, the most spoken second language was Spanish (64% of bilingual participants). Participants typically had varied educational backgrounds, with the majority having completed some college or an associate/bachelor’s degree (69%). Most were employed (60%) and reported an annual household income between $20,000 and $39,999 (73%). A significant majority relied on Medicaid or Medicare insurance (83%). Participants were commonly married or in an unmarried partnership (54%), while about one-third (34%) reported never being married. Nearly all participants cared for at least one dependent child (97%), or had 1t o 2 children (78%), most frequently aged between 6 and 12 years old (37%). Most participants (78%) reported themselves or their dependent having special healthcare needs. Participants also reported prior experience with chatbots (95%), including voice assistants like Siri and Alexa.

**Table 1:** Demographic characteristics of participants.

Category		N = 128	%
*Race*			
	White	56	44%
	Black / African American	46	36%
	Native American / American Indian	5	4%
	Mixed (selected 2 or more)	15	12%
	Unknown / Prefer not to answer	6	4%
*Ethnicity*			
	Hispanic or Latino	34	27%
	Non-Hispanic or Latino	94	73%
*Sex*			
	Female	87	68%
	Male	41	32%
*Age Groups of Participants*			
	18-29	25	20%
	30-39	53	41%
	40-49	39	30%
	50-66	11	9%
*Locations (U.S.)*			
	North	14	11%
	South	68	53%
	West	24	19%
	Midwest	22	17%
*Bilingual*			
	No	100	78%
	Yes*	28	22%
	Spanish	18	
	German	1	
	French	1	
	Prefer not to answer	8	
*English Fluency (of Bilinguals)*			
	Fluent	26	
	Good	1	
	Limited	1	
	None	0	
*Education*			
	Highschool (Diploma, GED)	27	21%
	Some college, no degree	41	32%
	Associate degree (AA, AS)	22	17%
	Bachelors (BA, BS, BBA, AB)	26	20%
	Masters (MA, MS, MEng, MEd, MBA)	9	7%
	Doctoral Degree (PhD, PsyD, EdD)	2	2%
	None of the above	1	1%
*Household Income*			
	Less than $10,000	19	14%
	$10,000 − $15,999	3	2%
	$16,000 − $19,999	12	9%
	$20,000 − $29,999	40	31%
	$30,000 − $39,999	54	42%
*Employment Status*			
	Currently Employed*	77	60%
	Early (0–2 years)	15	
	Mid (3–6 years)	26	
	Senior (7 + years)	36	
	Seeking Employment*	51	40%
	1 month or less	1	
	2-6 months	4	
	7 months to a year	4	
	Longer than a year	42	
*Health Insurance*			
	Medicare / Medicaid	107	83%
	Other Insurance Plan	5	4%
	Uninsured	16	13%
*Marital Status*			
	Married	47	37%
	Divorced	10	8%
	Widowed	1	1%
	Separated	4	3%
	Never married	44	34%
	A member of an unmarried couple	22	17%
*Child Dependents*			
	None	4	3%
	1-2	100	78%
	3-4	22	17%
	5 or more	2	2%
*Special Healthcare Needs (Self or Dependents)*			
	Yes	100	78%
	No	22	17%
	Prefer not to answer	2	2%
	Don’t have child dependents	4	3%
Age Groups of Dependents**			
	0-2 years old	26	
	3-5 years old	36	
	6-12 years old	61	
	13-17 years old	44	
	Prefer not to answer	0	
*Chatbot Use Experiences*			
	Don’t Use Chatbots	7	5%
	Yes*	121	95%
	< 2 times per week	38	
	2-6 times per week	44	
	> 7 times per week	39	

* Sub-group demographics do not include percentages to reduce misinterpretation across groups

** Not mutually exclusive, as participants could report more than one dependent

### Health-related social needs

Nearly all participants who engaged with the chatbot reported at least one unmet HRSN (98%). In Table 2, we provided combined scores (binary; y/n) of HRSNs grouped by short-term needs, long-term needs, and time-independent needs (full responses are available in Table A in S4 Appendix). The most frequently reported short-term (within the past 30 days) needs included food insecurity (75%), financial limitations (75%), and transportation barriers (57%). Food insecurity (71%) was also the most frequently reported long-term (within the past 12 months) need, followed by transportation barriers (48%) and financial limitations (38%). Additionally, while relatively few participants reported recent physical isolation from social activities (11%), a substantial number reported persistent feelings of social isolation (65%) without a specified time frame. Smaller proportions indicated difficulties related to overall literacy (reading and comprehension; 10%) and medical literacy confidence in managing health-related issues (9%).

**Table 2:** Frequencies of user-reported health-related social needs.

HRSNs		N = 128	%
*Short Term (30d need)*			
	Housing	59	46%
	Food	97	75%
	Transport	73	57%
	Finance	96	75%
	Isolation	14	11%
*Long Term (12m need)*			
	Housing	36	28%
	Food	91	71%
	Transport	62	48%
	Finance	49	38%
*Time-Independent*			
	Isolation	83	65%
	Literacy (overall)	13	10%
	Literacy (medical)	12	9%
*No HRSNs disclosed*		2	2%

### User experience

As reported in Table 3, participants rated the chatbot as being highly usable (mean = 84.7, SD = 12.4), trustworthy (mean = 4.1, SD = 0.8), and satisfactory (mean = 8.0, SD = 2.4). The chatbot was rated around the lowest third of the task load index (mean = 6.8, SD = 2.8).

**Table 3:** Descriptive results on user experience categories.

Characteristic	Task load	Satisfaction	Trust	Usability
Mean (SD)	6.8 (2.8)	8.0 (2.4)	4.1 (0.8)	84.7 (12.4)
Median (Q1, Q3)	6.3 (4.3, 8.8)	9.0 (7.0, 10.0)	4.0 (4.0, 5.0)	88.0 (78.0, 95.0)
Min, Max	1.8, 13.3	0.0, 10.0	2.0, 5.0	46.0, 100.0

### Exploratory analysis

We examined the association between demographic factors and user experience metrics using category-wise regression models (non-monotonic trends in S8 Appendix).

### Demographic factors and user experience

Perceived burden (Task Load) was significantly lower among participants who were Divorced (β = –2.7; 95% CI: –4.6, –0.85), Never Married (β = –1.3; 95% CI: –2.4, –0.14), and Unmarried (β = –1.4; 95% CI: –2.8, 0.00), relative to those who were Married. When aggregated, all non-married statuses were associated with significantly lower Task Load scores (β = –1.5; 95% CI: –2.5, –0.46). Satisfaction ratings were significantly higher among middle-aged participants compared to young adults (β = 0.99; 95% CI: 0.14, 1.8). No statistically significant associations were observed between usability and trust ratings vs. demographic variables. See Fig 3 for forest plots and Table B in S4 Appendix for regression results.

**Fig 3:**
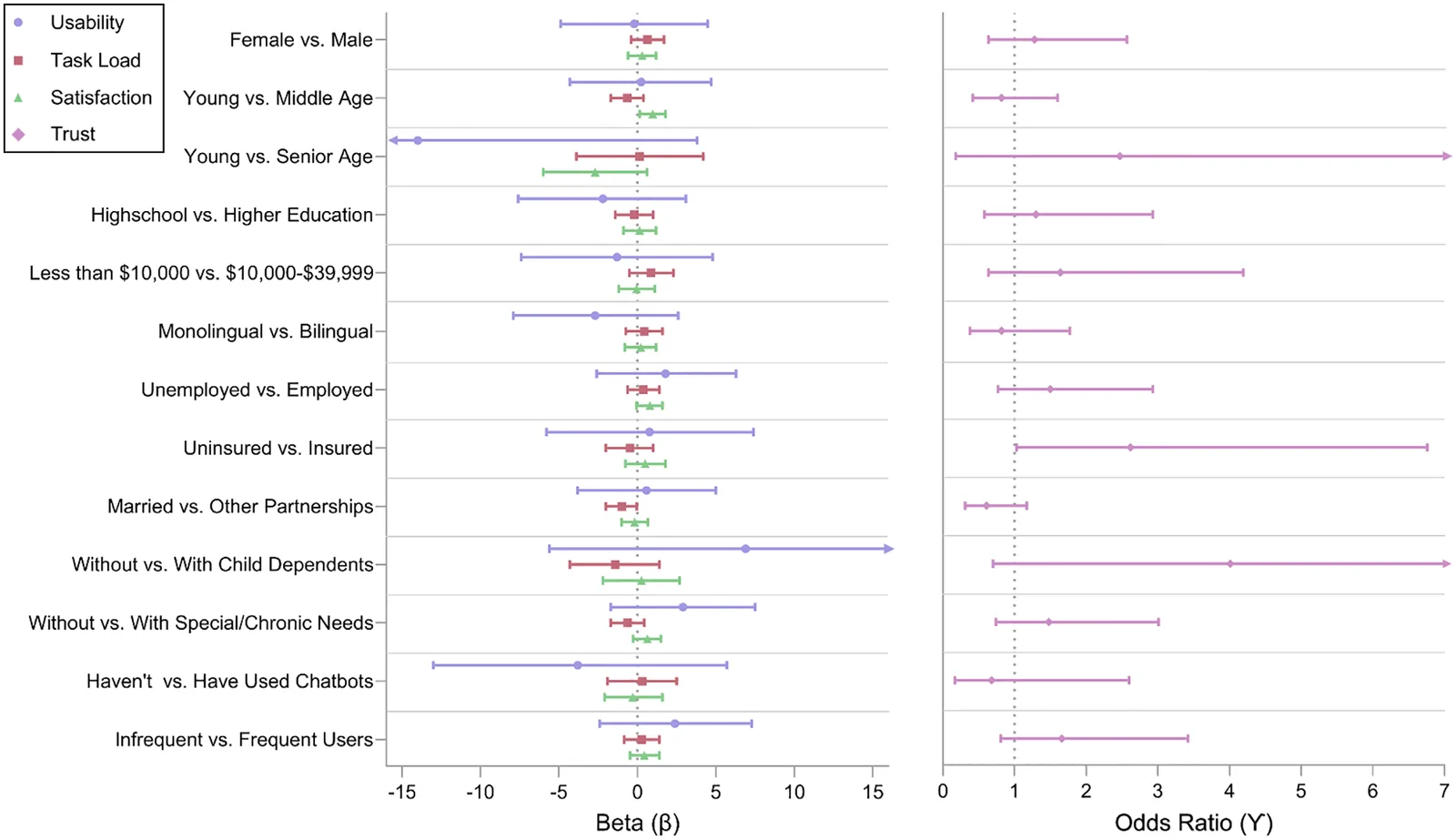
Regression analysis forest plot for demographics (binary) and user experience. First variable in each category is the reference value.

In terms of monotonic trends, most of the metrics scaled up with income from $10k onwards. Specifically, the most pronounced pairwise contrast ([$30k-$40k] – [$10k-$16k]) showed an estimated difference between the mean of Usability (Estimate = 8.85, SE = 7.40), Task Load (Estimate = 1.28, SE = 1.69), and Satisfaction (Estimate = 3.39, SE = 1.41), with differences scaling down as income decreased. It should be noted, incomes below $10k do not follow this trend. Likewise, a pronounced estimated difference was observed between ‘Masters degree’ - ‘Some college, no degree’ in terms of task load (Estimate = 3.17, SE = 1.00), with task load estimated difference scaling down as education scales down. Education at the high school level, however, did not follow this trend as it only scaled down to ‘Some college, no degree’. Suggestive trends are presented in Figs A-D in S8 Appendix; Tables A-J in S9 Appendix; and Tables A-C in S10 Appendix.

### Health related social needs and user experience

We examined associations between HRSNs and user experience metrics (see Fig 4 for forest plots; regression results are available in **Table C in** S4 Appendix **4**). Participants who reported that transportation issues prevented them from attending medical appointments had significantly higher trust ratings compared to those without such barriers (OR = 3.21; 95% CI: 1.03, 10.7). Users who reported being “very often worried about food” reported significantly lower task load scores than those who were never worried (β = –1.8; 95% CI: –3.6, –0.11). Participants who reported any type of transportation-related access issue (compared to those without any difficulty traveling) had significantly higher satisfaction scores (β = 0.89; 95% CI: 0.06, 1.7). No statistically significant associations were observed between HRSNs and usability.

**Fig 4:**
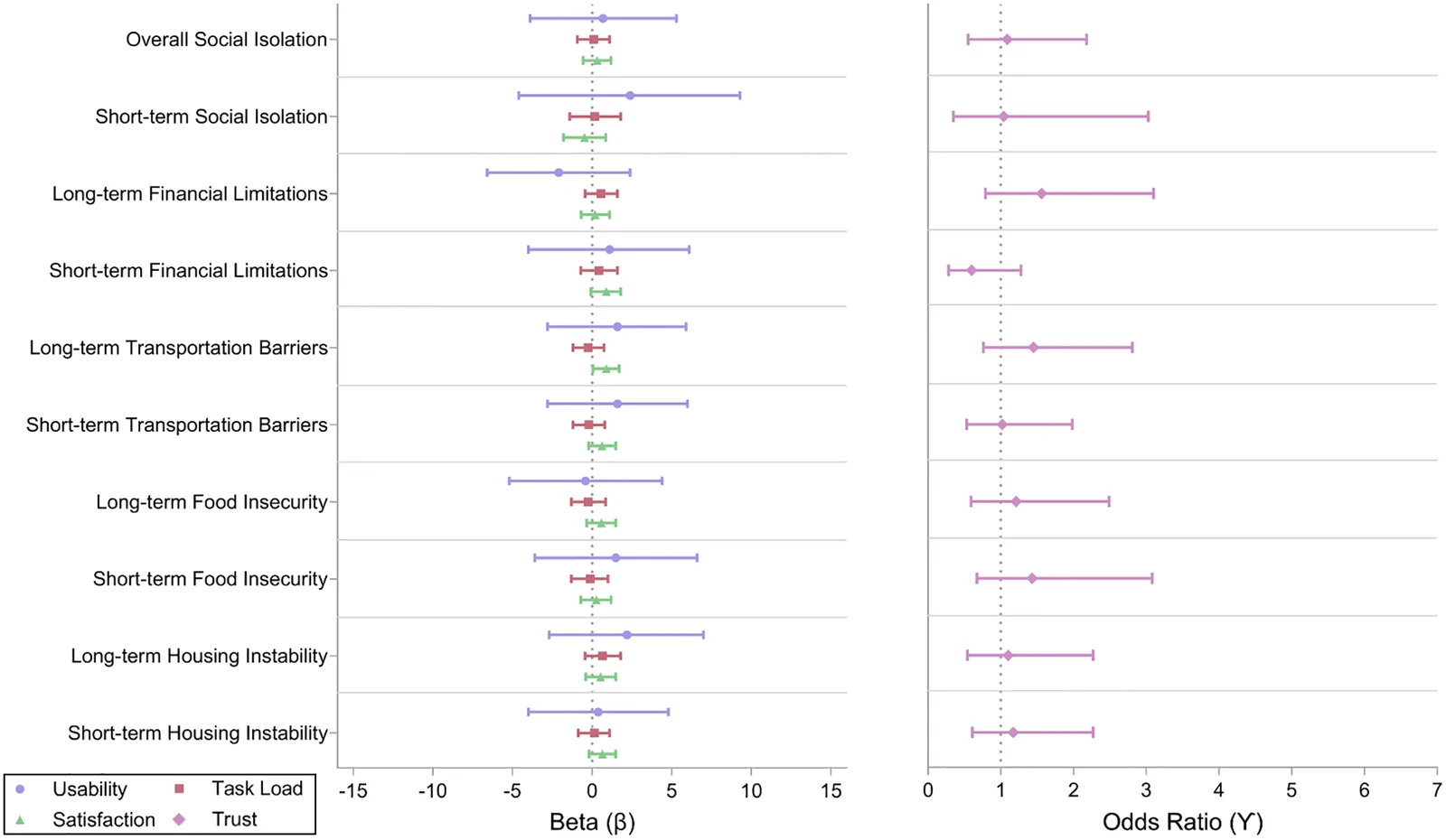
Regression analysis forest plot for HRSNs (binary) and user experience. First variable in each category is the reference value.

Several additional monotonic trends emerged that did not reach statistical significance but may warrant future exploration. The contrast for food insecurity (Often not enough to eat - Enough of the kinds of food we want to eat) showed a pronounced difference in estimated means for usability (Estimate = 9.59, SE = 5.18), and satisfaction (Estimate = 1.48, SE = 1.00), scaling down as food availability increased. Likewise, this contrast showed the most pronounced difference in task load (Estimate = -1.75, SE = 1.17), scaling up as food availability increased. Reports of utility shutoff followed a stepped pattern, with increasing levels of disruption (No → Yes → Already shut off) scaled positively with trust (Estimates for forest plots scaling up from 3.00 to 3.33). The contrast for affording utilities (Very Hard - Not hard at all) showed a pronounced difference in usability (Estimate = 1.72, SE = 3.34) and satisfaction (Estimate = 1.18, SE = 0.63), scaling down as difficulty decreased. Suggestive trends are presented in **Figs D-F in** **S8 Appendix****; Tables K-T in** **S9 Appendix****; and Tables D-G in** **S10 Appendix**.

### Conversational analytics

To better understand user interactions and preferences, we analyzed chatbot conversation transcripts, examining message characteristics, interaction types, content specificity, and conversation topics. Since the interactions for scenarios 1–3 are scripted, our analysis in this section is only focused on conversational data during the free engagement (last scenario). Participants generated a total of 204 chatbot transcripts through their engagements, where each user held between 1–4 unique conversations (M = 1.46, SD = 0.76).

Participants interacted with the chatbot for approximately 3 minutes per session (M = 3 min 8 sec, SD = 4 min 59 sec), averaging 20 conversational turns (range: 3–49). Individual user messages averaged 3 words (17 characters), ranging from brief inputs (1 word, 3 characters) to longer statements (up to 43 words, 214 characters). Participants strongly preferred using assistive buttons (N = 2,749 button presses) rather than free-text entries (N = 1,151), with the ‘Coverage’ button most frequently used prior to session completion (see Table A in S5 Appendix).

**Box 1. Example DAPHNE conversation**

DAPHNE: “Hey, I’m DAPHNE. If you need support with food, transportation, housing, or financial assistance, I’m here to help.”

Participant 2: “I’m really struggling with my diet.”

DAPHNE: “You’re not alone. Many people have trouble eating healthily because of cost or access. I could find something close to get you started. Would you mind sharing your zip code?

Participant 2: “#####”

DAPHNE: “Thanks for sharing! Good news, there’s a few food pantries in ##### that offer free groceries.” [lists 3 facilities]

Participant 2: [presses ‘Coverage’ button on facility 1 of 3]

DAPHNE: “This facility is open now and serves your area; typically, during weekdays from 9-5pm.”

Participant 2: [exits conversation]

Further qualitative examination of free-text entries provided deeper insights into how users communicated their resource needs. Using a structured rubric (S3 Appendix), two raters (AH and DIJ) assessed message specificity and user behaviors, achieving substantial agreement (Cohen’s Kappa; κ = 0.721). Users typically indicated specific community resources or facilities by name (M = 0.37, SD = 0.48), while a minority of the initiated interactions unrelated to social or resource needs (M = 0.91, SD = 0.28) (see Table B in S5 Appendix).

“I need essentials needs such as [food delivery service] and [city utilities] assistance.” (P109)

“Can you find arcades near [zipcode]?” (P80)

Keyword frequency analysis further illustrated user priorities (see **Table C** in S5 Appendix), highlighting terms closely aligned with resource needs such as ‘program(s),’ (16%) ‘shelter,’ (12%) and ‘food’ (8%). Conversation topics similarly reflected key user concerns, predominantly focusing on financial issues (18%), housing (17%), and nutritional (16%) needs. Users also raised diverse unique topics beyond predefined HRSN categories, underscoring the chatbot’s capacity to address a broad range of community-related issues (see Table D in S5 Appendix). Including fallbacks in the conversation, chatbot successfully identified the user’s intent 1511 out of 1523 messages (99.21%).

“Resources for [a parent] looking for a job” (P58)

“Delivered meals?” (P78)

“Where’s the nearest legal office?” (P26)

### User preferences

Participant free-text comments (open-ended responses to a positive experience, a negative experience, and suggested improvements for the chatbot) were analyzed and categorized into 5 qualitative domains: Knowledge, Delivery, Positive Feedback, Negative Feedback, and Suggestions (see S6 Appendix).

The Knowledge domain captured how participants approached learning about the recommended resource. In their free-text feedback, participants underscored a preference for direct links embedded within the chat for immediate call forwarding or archival purposes. Other users approached recommendations by comparing scheduling availability with the chatbot to determine the best fit relative to urgency. Some users engaged mostly with location-based customization to tailor recommendations by travel time.

“I can use the phone numbers or links to follow up later on this resource.” (P24)

“I want to make sure the resource I choose is open when I can get there.” (P95)

“If it’s too far, then I want to adjust the range like other apps do.” (P106)

The Delivery domain included specific features that were highlighted by participants. Users reinforced previous survey answers about chatbot usability, often noting the reasoning behind evaluation fit was a primary strength of the chatbot. Many appreciated the chatbot’s response speed after pivoting topics of interest. A subgroup of participants desired more ways to access the chatbot beyond the prototype, suggesting a voice-interaction plugin to further explain HRSNs and their impact on overall wellbeing.

“It kinda sorted out my needs for me.” (P91)

“It was seedy and quick in response to my many questions.” (P37)

“Maybe it could break it down for my by communicating via voice.” (P18)

Direct feedback on the chatbot was divided between Positive and Negative domains. Positive Feedback was defined by the chatbot’s ease of use and resource richness, consistently offering a variety of options when possible. Additionally, users who were unsure about what to explore highlighted the conversational tone might be a contributing factor for their positive experience when casually learning about other HRSNs. Sentiment analysis of the comments indicated generally positive feedback (M = 3.41, SD = 0.97), and intensity analysis showed moderate emotional expressiveness (M = 3.02, SD = 0.46). Despite this, Negative Feedback was defined by feature gaps or limitations in how features were implemented for conversational interaction. Participants noted a need for more relevant local options when considering multiple people’s needs concurrently. Other participants felt there was a gap in voice capabilities, as the chatbot could only reply with text-based responses.

“I like how it gave suggestions on info to look up.” (P9)

“The only negative is that the chatbot couldn’t ‘talk’ to me aloud.” (P88)

The Suggestion domain captured desirable future improvements to the chatbot for long term use. Participants most frequently wanted an improved mechanism to refine their search early in the conversation. This divided the users on their behavior, as some would have preferred a reduction in conversational turns for speed whereas others preferred support refining their goals in conversation. Other comments covered a need for targeting subgroups of adults when determining resource fit.

“It should help me reduce my queries to a question rather than many.” (P90)

“It should start by helping me be more specific when typing a question.” (P3)

“It could provide options that are better suited for young adults.” (P67)

After completion of the last scenario, nearly all participants (94%) reported the DAPHNE chatbot helpful in identifying relevant community resources. Post testing, they also reported preferred chatbot app enhancements (for future improvements beyond conversational capabilities). Participants expressed interest in several additional features, notably the inclusion of a “History and Favorites” (19%), the ability to create personal profiles (17%), password protection for enhanced security (15%), daily tips and personalized updates (15%), multilingual support (12%), integration with personal medical records (11%), and phone notifications (10%). A small proportion of users also suggested incorporating encouraging messaging, varied chatbot personalities, and additional technical guidance (see S7 Appendix).

## Discussion

The present mixed-methods evaluation sought to investigate user experience towards an AI-enabled chatbot (DAPHNE) that helped to identify unmet HRSNs and share real-time, community-based resource recommendations for the low-income earning cohort. Building on our prior development [18], we assessed the usability, task load, satisfaction, and trust of DAPHNE with qualitative analyses of conversational transcripts and free-text feedback. In doing so, we offer one of the first empirical demonstrations that a rules-guided, LLM-supported chatbot can deliver just-in-time HRSN guidance with high acceptability (in terms of usability, satisfaction and trust) and minimal cognitive burden (task load).

### Demographic composition and reported HRSNs

The study sample was largely representative of the racially and ethnically diverse, low-income, caregiver population targeted by current U.S. screening recommendations. Nearly all participants reported at least one unmet HRSN, with food insecurity and financial strain predominating in the short term. The co-occurrence of multiple needs, including transportation barriers and chronic social isolation, underscores the layered vulnerabilities confronting these families and highlights the importance of integrated referral mechanisms rather than siloed interventions [48,49]. Although online recruitment may select for digitally literate participants, the high prevalence of Medicaid coverage and bilingual status suggests that DAPHNE assessed by the cohort who are often underserved by traditional resource-navigation models [50,51].

### User experiences

Across standardized instruments, the chatbot demonstrated high usability, low task load, satisfaction, and substantial perceived trustworthiness -metrics that surpass typical digital health benchmarks and are notable given the complexity of HRSN navigation [51,52]. Conversational-analytics reinforce these quantitative ratings. Users interacted through succinct, goal-oriented exchanges via text or button options. The dialogue manager mapped intents with high fidelity, and fallback LLM prompts were rarely invoked, all of which enabled transparent dialogue and align with the low task load observed [53,54].

At the same time, qualitative feedback clarifies *what* users felt worked well and *what* they still needed. Participants frequently described the interface as intuitive and fast, reinforcing the high usability and satisfaction ratings. However, they also identified specific information gaps that constrain real-world usefulness, particularly requests for detailed eligibility criteria, embedded hyperlinks or direct call-forwarding, and finer geospatial granularity. Users further emphasized that broader accessibility features (e.g., voice input, personalized dashboards, secure logins, and proactive notifications) would make the tool more usable beyond the study context. Sentiment analysis suggested generally positive feedback but only moderate emotional engagement, suggesting that even with high acceptability, users may still expect more empathic and context-aware dialogue for sustained use [55–57] Calibrating empathic dialogue with the user’s expectations warrants further investigation [58,59]. Together, the quantitative ratings demonstrate strong acceptability and low burden, while the qualitative findings identify the specific design and content enhancements most likely to convert brief, low-friction interactions into longitudinal support [55].

### Relationship between demographics, HRSN and user experience

Demographic and HRSN covariates revealed limited but informative associations with user experience outcomes. Usability and trust were broadly consistent across sex [60], age [61], and insurance status, suggesting wide acceptability across populations that are often underserved in digital health contexts [62]. One of the clearest demographic patterns emerged in task load: non-married participants, including those who were divorced, never married, or unmarried, reported significantly lower task load compared to their married peers. This may reflect differences in prior experience independently coordinating needs or navigating digital tools, although the relationship between marital status and technology-related burden remains underexplored and warrants confirmation in future studies.

Several directional trends also help interpret variation in perceived usability and burden. Participants with lower income showed a trend toward reduced usability, while participants with higher education trended toward lower usability and higher task load. This pattern contrasts with common assumptions that education confers a consistent advantage for digital health engagement [63,64]. One plausible explanation is expectation calibration: in a uniformly low-income sample, higher education may raise expectations about specificity (e.g., eligibility details, and direct links). Therefore, limitations in the referral data or interaction flow may be judged more harshly, consistent with participants’ qualitative requests for deeper informational specificity and streamlined refinement of searches. Similarly, participants already familiar with established ways of finding resources (digital or traditional) may perceive the chatbot as redundant or inefficient (e.g., extra steps of conversations), contributing to a higher task load. These findings suggest the chatbot may be particularly beneficial for individuals who are new to navigating resource systems. This interpretation is consistent with findings from Kocielnik et al. [21], who reported that emergency department patients with lower health literacy were more receptive to chatbot-based screening than those with higher literacy levels.

HRSN-related patterns further emphasize that user experience depends on context, not just interface quality. Participants reporting transportation limitations or recent utility shutoffs (two direct indicators of material hardship) reported higher trust and satisfaction, suggesting that when conventional access is disrupted, the perceived utility of rapid, location-based navigation may increase. This interpretation consistent with evidence that resource-constrained users value tools that reduce friction in service navigation [65]. Users who reported being very often worried about food also reported lower task load, possibly suggesting that the chatbot was perceived as cognitively manageable during periods of stress, which is an important consideration given known links between food insecurity and reduced bandwidth for health engagement [66]. Notably, participants reporting limited social interaction (often used as a proxy for social isolation) showed a non-significant trend toward higher satisfaction, which, together with qualitative feedback about supportive conversational tone, adds to emerging evidence that chatbots may have perceived relational or emotional utility for some users [67–69]. These findings challenge the assumption that unmet needs uniformly impair digital engagement; instead, they suggest that digital tools may offer unique and sometimes amplified value for users navigating structural barriers [70].

### Implications

Identification for HRSNs in clinical settings is a crucial first step, though often does not translate into successful resource connections for patients out of the clinic [36,71]. Therefore, these results are encouraging. DAPHNE functioned not as a passive screener but as an active bridge to tailored referrals, addressing the long-standing “screen-to-referral” gap documented [72,73]. From a design perspective, our findings imply that a social-care chatbot should pursue quick, low-load button prompts while preserving free-text channels that capture nuanced circumstances. Equity-focused co-design involving community stakeholders and ongoing equity audits, tracking intent-recognition errors and unmet requests, will be critical to prevent digital access biases and safeguard equitable linkage to resources [74,75]. Moreover, because technology alone cannot resolve structural barriers, effective implementation requires provider-technology collaboration, user education, and deliberate positioning of chatbots as social-care navigators rather than standalone solutions. These strategies can help ensure culturally relevant and context-sensitive support, aligning with contemporary health equity frameworks [76].

Looking ahead, our study can initiate discussions on the potential for AI-enabled chatbots to support policy mandates for HRSNs screening, identification and requirements of resource sharing. Given current CMS requirements for hospitals to screen and report social drivers of health and Medicaid initiatives incentivizing systematic HRSN assessment [77], AI-enabled chatbots could substantially ease implementation burdens [78]. However, the United States Preventative Services Task Force has so far found insufficient evidence to recommend universal screening for certain social risks (such as food insecurity) due to uncertain impact on health outcomes [79]. This evidence gap highlights that simply implementing screening mandates is not enough, effective solutions are needed to actually address the identified needs. Our findings contribute to an emerging evidence base by illustrating that an AI-enabled chatbot, as a navigator, can achieve high engagement and potentially assist in resource connection. If such tools can be proven to improve outcomes (for example, reducing unmet needs or downstream health care utilization), they could substantially strengthen the case for routine social risk identification and intervention as a covered, reimbursable preventive service [80]. However, DAPHNE is not an autonomous platform. DAPHNE was designed to function within the ecosystem of public health and services. Therefore, it depends on available and up-to-date local, regional or national community resources that are routinely curated and maintained by public health and social care service providers.

### Strengths and limitations

Key strengths of this study include the mixed-methods, convergent design and deployment of the chatbot in a realistic context with real-time resource data. Collecting quantitative (survey) and qualitative (interaction logs and free-text questionnaires) data allowed us to triangulate findings, enhancing the validity of our conclusions [81]. Sentiment analysis better modelled the value of empathetic language [82] than quantitatively enforcing certain chatbot replies [83] similarly found in other studies of human-computer interaction [58,59]. The use of an actual resource referral API (as opposed to a static or hypothetical list) increases the validity of our usability results and demonstrates technical feasibility for real-world integration. As the chatbot, one of DAPHNE’s key advantages over general-purpose public chatbots is its purpose-built, constrained design, which prioritizes accuracy of local resource information, low cognitive burden, and appropriate framing, rather than broad open-ended generation. By grounding responses in verified community resource data and limiting generative flexibility, DAPHNE is designed to support trustworthy, context-specific navigation in ways that general-purpose AI tools are not optimized to provide. We also prioritized user-centered and equitable development, recruiting a diverse sample of participants and incorporating their feedback into future design refinements.

Nonetheless, several limitations warrant discussion. First, outcomes were measured via self-report instruments and short-term task performance in English language only; we did not directly measure long-term impacts on resource uptake or health outcomes including multiple languages. Future work on DAPHNE and other patient-facing conversational agents could seek to mitigate language discordance by engaging in a user’s preferred reading or spoken language [84]. Health literacy was captured within 2 self-reported items (See S4 Appendix) compared to in-person studies within healthcare [85–89] which could be expanded to measure how literacy impacts chatbot use. Participants’ subjective ratings of trust and satisfaction may have been influenced by social desirability or novelty effects. Second, the study lacked a comparator group, without a control condition (e.g., usual care or an alternative navigation method), we cannot definitively attribute benefits to the chatbot or assess its relative effectiveness. Future studies should consider randomized trials or quasi-experimental designs to compare chatbot-assisted social care navigation against standard practice. Third, our sample size and setting limit generalizability. All participants had at least some internet access and consented virtually to engage with a digital tool; thus, our findings may not extend to those with severe digital literacy barriers or distrust of technology [90,91]. This digital divide bias means additional strategies are needed to reach and support the most marginalized groups who might not engage with a chatbot [92]. Fourth, chatbot was capable of providing guidance only based on single API based resource, and nuanced or complex HRSNs and guidance (such as, guidance to enroll support programs) may be lacking and affected perceived value. Fifth, we did not investigate human behavior against grounded theoretical behavioral models or frameworks. Sixth, we did not investigate participant perceptions on chatbot against human-based social support. Seventh, we excluded any extended cases, such as, services in high-risk cases (urgent needs), therapeutic interventions for mental health related social needs or additional expected features as voice interactions. Future works should be planned to investigate iteratively such approaches and cases. Finally, while our RAG-based approach was intended to ensure accuracy, we did not formally evaluate the accuracy of chatbot responses or recommendations. There remains a risk of hallucinations or errors when large language models are involved [93]. Although no serious issues were reported in our study, establishing rigorous content validation and oversight (such as human review of a subset of chatbot outputs or user-friendly feedback mechanisms) will be important as the system scales.

## Conclusions

In conclusion, our study demonstrates the promise of an AI-enabled chatbot, DAPHNE, to identify HRSNs and connect families with community resources in an acceptable and user-friendly manner. It contributes to the growing literature on digital social care practices and integration by providing empirical evidence that a chatbot can engage vulnerable populations with high usability, trust and satisfaction and low burden. To fully realize this potential, future work must rigorously evaluate effectiveness, ensure equity in design and deployment, and address practical implementation challenges. With continued refinement and evidence generation, AI-driven chatbots could become a valuable adjunct in the ongoing efforts to integrate social care into healthcare.

## Supporting information

S1 Appendix High-priority health-related social need scenarios. (DOCX)

S2 Appendix Rubric for sentiment and intensity. (DOCX)

S3 Appendix Rubric for specificity and behaviors. (DOCX)

S4 Appendix Self-reported health-related social needs and exploratory results. Table A. Self-reported health-related social needs. Table B. Statistical regressions between demographics and user experience. Table C. Statistical regressions between health-related social needs and user experiences.
(DOCX)

S5 Appendix Frequency of specific user behaviors. Table A. Frequency of assistive buttons before leaving a chat session. Table B. Patterns in resource-seeking behaviors with specificity. Table C. Frequencies of keywords in free text messages. Table D. Topics of interest.
(DOCX)

S6 Appendix Qualitative domains and themes. (DOCX)

S7 Appendix Chatbot helpfulness and desired future features. (DOCX)

S8 Appendix Forest plots of demographics, usability, and health-related social needs. Fig A. Demographics and Usability Forest Plot. Fig B. Demographics and Trust Forest Plot. Fig C. Demographics and Task Load Forest Plot. Fig D. Demographics and Satisfaction Forest Plot. Fig E. Health-related Social Need and Usability Forest Plot. Fig F. Health-related Social Need and Trust Forest Plot. Fig G. Health-related Social Need and Task Load Forest Plot. Fig H. Health-related Social Need and Satisfaction Forest Plot.
(DOCX)

S9 Appendix Pairwise comparisons between demographics, usability, and health-related social needs. Table A. Education and Usability Pairwise Comparison. Table B. Income and Usability Pairwise Comparison. Table C. Children and Usability Pairwise Comparison. Table D. Education and Task Load Pairwise Comparison. Table E. Income and Task Load Pairwise Comparison. Table F. Children and Task Load Pairwise Comparison. Table G. Education and Satisfaction Pairwise Comparison. Table H. Income and Satisfaction Pairwise Comparison. Table I. Children and Satisfaction Pairwise Comparison. Table J. Food Quantity and Usability Pairwise Comparison. Table K. Utility Shutoff and Usability Pairwise Comparison. Table L. Needs Affordability and Usability Pairwise Comparison. Table M. Social Isolation and Usability Pairwise Comparison. Table N. Food Quantity and Task Load Pairwise Comparison. Table O. Utility Shutoff and Task Load Pairwise Comparison. Table P. Needs Affordability and Task Load Pairwise Comparison. Table Q. Social Isolation and Task Load Pairwise Comparison. Table R. Food Quantity and Satisfaction Pairwise Comparison. Table S. Utility Shutoff and Satisfaction Pairwise Comparison. Table T. Needs Affordability and Satisfaction Pairwise Comparison. Table U. Social Isolation and Satisfaction Pairwise Comparison.
(DOCX)

S10 Appendix Class estimates of demographics, usability, and health-related social needs. Table A. Education Mean Trust Class Estimate. Table B. Income Mean Trust Class Estimate. Table C. Kids Mean Trust Class Estimate. Table D. Food Quantity Mean Trust Class Estimate. Table E. Utility Shutoff Mean Trust Class Estimate. Table F. Needs Affordability Mean Trust Class Estimate. Table G. Social Isolation Mean Trust Class Estimate.
(DOCX)

S11 Appendix Large language model system prompts and parameters. (DOCX)

S1 Checklist STROBE checklist. (DOCX)
