# Supplementary material for: Evaluating user experiences with an AI chatbot for health-related social needs: A cross-sectional mixed methods study
Source: PLOS Digit Health. 2026 Oct 30;5(10):e0001789. doi: 10.1371/journal.pdig.0001789 (PMC13722182; doi:10.1371/journal.pdig.0001789)
Supplement: Supplementary file 1 [file pdig.0001789.s001.docx]

# **S1 Appendix.** High priority health-related social need scenarios

## Scenario 1 of 4: Finding a food program

In this scenario 1, let's assume you need to find currently available nutrition support programs:

1. In response to the chatbot's question ("What are you looking for today?"), ask about a nutrition program (e.g., food pantries, soup kitchens, or home-delivered meals)
2. Type the zip code: 43201 into the chatbot and confirm.
3. Review suggested 3 resources and programs.
4. Read "More Details" of one of the programs suggested.
5. Check operating "Business Hours" of the program you selected.

## Scenario 2 of 4: Giving feedback & learning cost

In this scenario 2, we will change the conversation towards finding health related resources:

1. Continue the previous conversation by clicking "I'm Done."
2. When asked if the latest recommendation helped, select "Yes". This feedback will be used to update our resources and improve our responses for our users the next time.
3. Then, the chatbot asks if any other help is needed, click or type "Yes." to ask for other resources.
4. Select or type for "health-related" resources and then enter new zip code: 43215
5. Expand the suggestions by clicking on "More Resources"
6. Pick one of the resources or programs suggested by clicking "More Details" and read the information.
7. Ask about the program's "Cost" and read the information.
8. Click on "Go Back" to list again the resources.

## Scenario 3 of 4: Housing

In this scenario 3, we will explore housing-related supporting programs:

1. Continue the previous conversation and click on "Change My Location"
2. Enter zip code: 45402 and review the suggested resources.
3. Click on "Find Help for Something Else" and type to ask about shelter and residential programs.
4. Click on "Contact Info" of any suggested program and review contact information, and then ask about their "Coverage."

## Scenario 4 of 4: Exploration

In this scenario 4, you will be able to use the chatbot as you prefer:

1. Click on "I'm Done", then follow conversation to agree or disagree that you found what you were looking for, and then click "Yes" to the question of "Is there anything else I can help with?"
2. In this new session, explore the chatbot freely. Search for any community resources for yourself or someone you know with any zip code.
   - You may compare programs with other nearby zip codes.
   - You may try viewing the location of a resource using the "Open Maps" button.
