# Supplementary material for: Evaluating user experiences with an AI chatbot for health-related social needs: A cross-sectional mixed methods study
Source: PLOS Digit Health. 2026 Oct 30;5(10):e0001789. doi: 10.1371/journal.pdig.0001789 (PMC13722182; doi:10.1371/journal.pdig.0001789)
Supplement: Supplementary file 2 [file pdig.0001789.s002.docx]

# **S2 Appendix.** Rubric for Sentiment and Intensity

**Sentiment Category (1–5)**

- **1 (Negative)**: Strong dissatisfaction, frustration, confusion, disappointment, or harsh criticism
- **2 (Somewhat Negative)**: Mild dissatisfaction, frustration, or confusion
- **3 (Neutral)**: Factual statements without distinct emotion; balanced or mixed with little to no affect
- **4 (Somewhat Positive)**: Mild satisfaction or generally positive feelings
- **5 (Positive)**: Strong satisfaction, appreciation, happiness, or clear success

**Intensity Level (1–5)**

- **1 (Low)**: Minimal or negligible emotional content
- **2 (Moderately Low)**: Mild emotional content, somewhat subtle expressions of sentiment
- **3 (Medium)**: Moderate emotional expression, neither extreme nor minimal
- **4 (Moderately High)**: Noticeable emotional language but not overwhelmingly strong
- **5 (High)**: Very strong emotional language, clearly intense (e.g., “extremely,” “fantastic,” “terrible”)

**Application Guidelines**

1. **Provide Two Ratings:**

For each user response, give both a “Sentiment Category” rating (1–5) and an “Intensity Level” rating (1–5), along with a brief explanation.

2. **Handling Borderline Cases:**

If it’s unclear whether to classify feedback as 1 (Negative) or 2 (Somewhat Negative), or 4 (Somewhat Positive) or 5 (Positive), lean toward 2 or 4 unless the feedback clearly belongs in the most negative or most positive category.

Use 3 (Neutral) for factual, balanced statements or moderate emotional expression.

3. **Mixed/Conflicting Sentiment:**

If a response shows both positive and negative aspects with different intensities, select the **more extreme** sentiment category (e.g., if a user mostly praises but includes one strongly critical point, the negative might dominate if it’s clearly intense).
