# Supplementary material for: Evaluating user experiences with an AI chatbot for health-related social needs: A cross-sectional mixed methods study
Source: PLOS Digit Health. 2026 Oct 30;5(10):e0001789. doi: 10.1371/journal.pdig.0001789 (PMC13722182; doi:10.1371/journal.pdig.0001789)
Supplement: Supplementary file 3 [file pdig.0001789.s003.docx]

# **S3 Appendix.** Rubric for Specificity and Behaviors

Score each category as either:

- 1 (Present): The feature is clearly included
- 0 (Absent): The feature is missing or too vague

Use the user’s message and chatbot prompt as context for evaluation.

### (1) Mentions of a Specific Resource

Score 1 if the participant names something more precise than a general category

Examples:

- Specific organizations or programs (e.g., “Medicaid”, “Salvation Army”)
- Subsets or qualifiers (e.g., “allergen-free food”, “bus tokens”, “computer classes”)

Score 0 if the response is generic or vague

Examples:

- “food help”
- “housing support”

Synonyms that restate a broad domain without narrowing it

Generic domain list: food, housing, goods, transit, health, money, care, education, work, legal

### (2) Mentions of the Purpose for the Resource

Score 1 if the user explains who the help is for or why they need it

Examples:

- “for my fiancée”
- “so I can get to work”
- “for my friend who’s diabetic”

Score 0 if there’s no purpose or only a vague plea

Example: “I just need help”

### (3) Mentions their Personal Background

Score 1 if the user shares any detail about their life situation

Examples:

- “I just moved”
- “I have 3 kids”
- “I lost my job”

Score 0 if there's no personal context provided.

### (4) Mentions Multiple Needs

Score 1 if the user identifies two or more distinct needs that require different types of assistance

Examples:

- From different domains (e.g., food and housing), or
- From the same domain but covering different timescales or scopes (e.g., groceries today vs. long-term SNAP)

Score 0 if only one need is mentioned, or multiple phrases point to the same issue

Examples:

- “place to stay” + “safe housing”
- “rent help” + “finding an apartment”

### (5) Mentions Thoughts on Finding a Resource

Score 1 if the user shows any interest in getting help or locating a resource

Examples:

- “I’m overwhelmed and don’t know what to do”
- “Is there a place I can go?”

Score 0 if the message is unrelated to help-seeking

Examples:

- Jokes
- Meta-comments (“This app is weird”)
- Disengagement (“idk”)
