# Supplementary material for: Evaluating user experiences with an AI chatbot for health-related social needs: A cross-sectional mixed methods study
Source: PLOS Digit Health. 2026 Oct 30;5(10):e0001789. doi: 10.1371/journal.pdig.0001789 (PMC13722182; doi:10.1371/journal.pdig.0001789)
Supplement: Supplementary file 4 [file pdig.0001789.s004.docx]

# **S4 Appendix.** Self-reported health-related social needs and exploratory results

## Table A. Self-reported health-related social needs

| **Health-related Social Needs** | | **N** | **%** |
| --- | --- | --- | --- |
| *Within the past 12 months, have you ever stayed: outside, in a car, in a tent, in an overnight shelter, or temporarily in someone else’s home (i.e. couch-surfing)?* | | | |
|  | Yes* | 36 | 28% |
|  | No | 92 | 72% |
| *In the last month, have you had concerns about the condition or quality of your housing?* | | | |
|  | Yes* | 59 | 46% |
|  | No | 69 | 54% |
| *Within the past 12 months, have you worried that your food would run out before you got money to buy more?* | | | |
|  | Never | 37 | 29% |
|  | Sometimes* | 58 | 45% |
|  | Often* | 19 | 15% |
|  | Very Often* | 14 | 11% |
| *Which of these statements best describes the food eaten in your household in the last month?* | | | |
|  | Often not enough to eat* | 7 | 5% |
|  | Sometimes not enough to eat* | 17 | 13% |
|  | Enough but not always the kinds of food we want* | 73 | 57% |
|  | Enough of the kinds of food we want to eat | 31 | 25% |
| *In the past 12 months, has lack of transportation kept you from medical appointments, meetings, work, or from getting things needed for daily living?* ******** | | | |
|  | Yes, it has kept me from medical appointments or getting medications* | 34 | 27% |
|  | Yes, it has kept me from non-medical meetings, appointments, work, or getting things that I needed* | 48 | 38% |
|  | No | 66 | 52% |
| *In the last month, did you put off or neglect going somewhere because of distance or transportation?* | | | |
|  | Yes* | 73 | 57% |
|  | No | 55 | 43% |
| *In the past 12 months, has the electric, gas, oil, or water company threatened to shut off services in your home?* | | | |
|  | No | 79 | 62% |
|  | Yes* | 44 | 34% |
|  | Already Shut Off* | 5 | 4% |
| *In the last month, how hard was it for you to pay for the very basics like food, housing, medical care, and heating?* | | | |
|  | Not hard at all | 32 | 25% |
|  | Somewhat Hard* | 71 | 55% |
|  | Very hard* | 25 | 20% |
| *How often do you need to have someone help you when you read instructions, pamphlets, or other written material?* | | | |
|  | Never | 102 | 80% |
|  | Rarely | 13 | 10% |
|  | Sometimes* | 11 | 8% |
|  | Often* | 1 | 1% |
|  | Always* | 1 | 1% |
| *How confident are you filling out medical forms by yourself?* | | |  |
|  | Extremely | 82 | 64% |
|  | Very much | 34 | 27% |
|  | Slightly* | 9 | 7% |
|  | Slightly not* | 0 | 0% |
|  | Very little* | 2 | 2% |
|  | Not at all* | 1 | 1% |
| *How often do you feel isolated from others?* | |  |  |
|  | Hardly ever | 45 | 35% |
|  | Some of the time* | 49 | 38% |
|  | Often* | 34 | 27% |
| *How often do you see or talk to people that you care about and feel close to? (For example: Talk to friends over the phone, visiting friends or family, going to church or club meetings)* | | | |
|  | 5 or more time a week | 44 | 34% |
|  | 3 to 5 times a week | 34 | 27% |
|  | 1 or 2 times a week | 36 | 28% |
|  | Less than once a week* | 14 | 11% |
|  | ******* *Marked answers are positive screenings of HRSNs for binary scoring*  ******** *Not mutually exclusive, as multiple transportation needs were captured here* |  |  |

## Table B. Statistical regressions between demographics and user experience

|  | **Usability** | | **Trust** | | **Task load** | | **Satisfaction** | |
| --- | --- | --- | --- | --- | --- | --- | --- | --- |
| **Characteristic** | **Beta** | **95% CI** | **OR** | **95% CI** | **Beta** | **95% CI** | **Beta** | **95% CI** |
| **Sex** |  |  |  |  |  |  |  |  |
| Female | 0.00 | — | 1.00 | — | 0.00 | — | 0.00 | — |
| Male | -0.20 | -4.9, 4.5 | 1.28 | 0.64, 2.57 | 0.65 | -0.40, 1.7 | 0.31 | -0.59, 1.2 |
| **Age** |  |  |  |  |  |  |  |  |
| Young Adults | 0.00 | — | 1.00 | — | 0.00 | — | 0.00 | — |
| Middle-Aged Adults | 0.25 | -4.3, 4.7 | 0.82 | 0.42, 1.60 | -0.64 | -1.7, 0.39 | 0.99 | 0.14, 1.8 |
| Senior Adults | -14 | -31, 3.8 | 2.47 | 0.18, 59.5 | 0.15 | -3.9, 4.2 | -2.7 | -6.0, 0.63 |
| **Education** |  |  |  |  |  |  |  |  |
| Highschool | 0.00 | — | 1.00 | — | 0.00 | — | 0.00 | — |
| *Higher Education** | *-2.2* | *-7.6, 3.1* | *1.3* | *0.58, 2.93* | *-0.21* | *-1.4, 1.0* | *0.15* | *-0.88, 1.2* |
| Some college, no degree | 1.0 | -5.0, 7.1 | 1.89 | 0.75, 4.82 | -1.3 | -2.6, 0.06 | 0.67 | -0.51, 1.9 |
| Associate degree | -3.7 | -11, 3.3 | 1.06 | 0.37, 3.04 | -0.32 | -1.9, 1.2 | -0.30 | -1.7, 1.1 |
| Bachelor’s degree | -5.9 | -13, 0.79 | 0.90 | 0.32, 2.54 | 0.89 | -0.58, 2.4 | -0.31 | -1.6, 1.0 |
| Master’s degree | -4.9 | -14, 4.5 | 1.19 | 0.27, 5.21 | 1.9 | -0.16, 4.0 | -0.11 | -1.9, 1.7 |
| Other | 3.6 | -11, 18 | 0.98 | 0.10, 11.1 | -0.83 | -4.1, 2.4 | 1.1 | -1.8, 4.0 |
| **Income** |  |  |  |  |  |  |  |  |
| Less than $10,000 | 0.00 | — | 1.00 | — | 0.00 | — | 0.00 | — |
| *$10,000-$39,000** | -1.3 | -7.4, 4.8 | 1.64 | 0.64, 4.19 | 0.88 | -0.50, 2.3 | -0.05 | -1.2, 1.1 |
| $10,000-$19,999 | -5.0 | -13, 3.5 | 0.80 | 0.22, 2.91 | 0.52 | -1.4, 2.5 | -0.79 | -2.4, 0.85 |
| $20,000-$29,999 | -2.2 | -9.1, 4.6 | 2.03 | 0.71, 5.90 | 0.97 | -0.60, 2.5 | -0.30 | -1.6, 1.0 |
| $30,000-$39,999 | 0.40 | -6.2, 7.0 | 1.74 | 0.64, 4.74 | 0.92 | -0.57, 2.4 | 0.34 | -0.93, 1.6 |
| **Do you speak a language other than English at home?** |  |  |  |  |  |  |  |  |
| No | 0.00 | — | 1.00 | — | 0.00 | — | 0.00 | — |
| Yes | -2.7 | -7.9, 2.6 | 0.82 | 0.38, 1.77 | 0.44 | -0.75, 1.6 | 0.22 | -0.80, 1.2 |
| **Currently employed** |  |  |  |  |  |  |  |  |
| No | 0.00 | — | 1.00 | — | 0.00 | — | 0.00 | — |
| Yes | 1.8 | -2.6, 6.3 | 1.50 | 0.77, 2.93 | 0.38 | -0.63, 1.4 | 0.80 | -0.05, 1.6 |
| **Insurance** |  |  |  |  |  |  |  |  |
| No | 0.00 | — | 1.00 | — | 0.00 | — | 0.00 | — |
| Yes | 0.77 | -5.8, 7.4 | 2.62 | 1.03, 6.76 | -0.46 | -2.0, 1.0 | 0.51 | -0.76, 1.8 |
| **Marital Status** |  |  |  |  |  |  |  |  |
| Married | 0.00 | — | 1.00 | — | 0.00 | — | 0.00 | — |
| *Other Partnerships** | *2.8* | *-1.7, 7.3* | *0.89* | *0.45, 1.74* | *-1.5* | *-2.5, -0.46* | *0.01* | *-0.86, 0.89* |
| Divorced | 5.7 | -2.8, 14 | 0.51 | 0.15, 1.78 | -2.7 | -4.6, -0.85 | 0.60 | -1.1, 2.3 |
| Widowed | -17 | -42, 7.9 | 0.09 | 0.00, 2.86 | 0.63 | -4.9, 6.1 | -3.0 | -7.8, 1.8 |
| Separated | 2.6 | -10, 15 | 0.72 | 0.11, 5.04 | -0.78 | -3.6, 2.1 | 0.25 | -2.2, 2.7 |
| Never Married | 1.7 | -3.5, 6.8 | 0.81 | 0.37, 1.76 | -1.3 | -2.4, -0.14 | -0.20 | -1.2, 0.80 |
| Unmarried Couple | 4.7 | -1.6, 11 | 1.65 | 0.63, 4.43 | -1.4 | -2.8, 0.00 | 0.27 | -0.96, 1.5 |
| **Current number of children under your care** |  |  |  |  |  |  |  |  |
| None | 0.00 | — | 1.00 | — | 0.00 | — | 0.00 | — |
| *Any Child Dependents** | *6.9* | *-5.6, 19* | *4.01* | *0.70, 22.9* | *-1.4* | *-4.3, 1.4* | *0.27* | *-2.2, 2.7* |
| 1-2 | 6.6 | -6.0, 19 | 3.86 | 0.67, 22.2 | -1.4 | -4.2, 1.5 | 0.14 | -2.3, 2.6 |
| 3-4 | 7.5 | -5.9, 21 | 4.53 | 0.68, 30.1 | -1.7 | -4.8, 1.4 | 0.66 | -1.9, 3.2 |
| 5 or more | 16 | -5.3, 37 | 10.2 | 0.47, 33.9 | -1.3 | -6.2, 3.5 | 2.3 | -1.9, 6.4 |
| **Do you or your dependent child have special** **healthcare needs or chronic conditions?** |  |  |  |  |  |  |  |  |
| No | 0.00 | — | 1.00 | — | 0.00 | — | 0.00 | — |
| Yes | 2.9 | -1.7, 7.5 | 1.48 | 0.74, 3.01 | -0.62 | -1.7, 0.43 | 0.64 | -0.26, 1.5 |
| **Prior use of chatbots or virtual assistants** |  |  |  |  |  |  |  |  |
| No | 0.00 | — | 1.00 | — | 0.00 | — | 0.00 | — |
| Yes | -3.8 | -13, 5.7 | 0.68 | 0.17, 2.60 | 0.32 | -1.9, 2.5 | -0.29 | -2.1, 1.6 |
| **Frequency of chatbot or virtual assistant use** |  |  |  |  |  |  |  |  |
| Infrequent (<2 times per week) | 0.00 | — | 1.00 | — | 0.00 | — | 0.00 | — |
| *Frequent (≥2 times per week)** | *2.4* | *-2.4, 7.3* | *1.66* | *0.81, 3.42* | *0.28* | *-0.83, 1.4* | *0.45* | *-0.47, 1.4* |
| Occasional (2-6 times per week) | 3.5 | -2.0, 9.0 | 1.93 | 0.85, 4.43 | -0.29 | -1.5, 0.95 | 0.66 | -0.39, 1.7 |
| Often (>7 times per week) | 1.3 | -4.4, 6.9 | 1.41 | 0.61, 3.25 | 0.94 | -0.34, 2.2 | 0.21 | -0.86, 1.3 |
| Abbreviations: CI = Confidence Interval, OR = Odds Ratio  * = Binarized comparison of means and confidence intervals between the reference point and all other possible characteristics per measure | | | | | | | | |

## Table C. Statistical regressions between health-related social needs and user experiences

|  | **Usability** | | **Trust** | | **Task load** | | **Satisfaction** | |
| --- | --- | --- | --- | --- | --- | --- | --- | --- |
| **Characteristic** | **Beta** | **95% CI** | **OR** | **95% CI** | **Beta** | **95% CI** | **Beta** | **95% CI** |
| **Within the last 12 months, have you ever stayed outside,** **in a car/tent/overnight shelter, or temporarily in someone else's home?** |  |  |  |  |  |  |  |  |
| No | 0.00 | — | 1.00 | — | 0.00 | — | 0.00 | — |
| Yes | 2.2 | -2.7, 7.0 | 1.10 | 0.54, 2.27 | 0.66 | -0.44, 1.8 | 0.53 | -0.40, 1.5 |
| **In the last month, any concerns about quality of housing?** |  |  |  |  |  |  |  |  |
| No | 0.00 | — | 1.00 | — | 0.00 | — | 0.00 | — |
| Yes | 0.39 | -4.0, 4.8 | 1.17 | 0.61, 2.27 | 0.14 | -0.86, 1.1 | 0.65 | -0.19, 1.5 |
| **Within the last 12 months, have you worried that your food** **would run out before you got money to buy more?** |  |  |  |  |  |  |  |  |
| No | 0.00 | — | 1.00 | — | 0.00 | — | 0.00 | — |
| *Yes** | *-0.42* | *-5.2, 4.4* | *1.21* | *0.59, 2.49* | *-0.25* | *-1.3, 0.85* | *0.58* | *-0.34, 1.5* |
| Sometimes | -0.63 | -5.9, 4.6 | 1.36 | 0.62, 2.96 | -0.07 | -1.2, 1.1 | 0.46 | -0.54, 1.5 |
| Often | -1.3 | -8.3, 5.7 | 0.75 | 0.26, 2.15 | 0.40 | -1.2, 2.0 | 0.30 | -1.0, 1.6 |
| Very Often | 1.6 | -6.2, 9.4 | 1.44 | 0.48, 4.43 | -1.8 | -3.6, -0.11 | 1.5 | -0.01, 3.0 |
| **Quantity of food eaten in your household within the last month:** |  |  |  |  |  |  |  |  |
| Enough of the kinds of food we want to eat | 0.00 | — | 1.00 | — | 0.00 | — | 0.00 | — |
| *Not enough or kinds of food** | *1.5* | *-3.6, 6.6* | *1.43* | *0.67, 3.08* | *-0.11* | *-1.3, 1.0* | *0.27* | *-0.72, 1.2* |
| Enough but not always the kinds of food we want | 0.59 | -4.7, 5.8 | 1.36 | 0.61, 3.01 | 0.22 | -0.97, 1.4 | 0.06 | -0.96, 1.1 |
| Sometimes not enough to eat | 2.0 | -5.4, 9.4 | 2.32 | 0.75, 7.42 | -0.87 | -2.5, 0.81 | 0.66 | -0.77, 2.1 |
| Often not enough to eat | 9.6 | -0.66, 20 | 0.98 | 0.25, 3.93 | -1.7 | -4.1, 0.58 | 1.5 | -0.50, 3.5 |
| **Has lack of transportation impacted your ability to get places?** |  |  |  |  |  |  |  |  |
| No | 0.00 | — | 1.00 | — | 0.00 | — | 0.00 | — |
| *Yes** | *1.6* | *-2.8, 5.9* | *1.45* | *0.76, 2.81* | *-0.25* | *-1.2, 0.74* | *0.89* | *0.06, 1.7* |
| Yes, both medical and non-medical appointments | -0.01 | -6.3, 6.3 | 1.04 | 0.42, 2.63 | -0.59 | -2.0, 0.85 | 1.1 | -0.08, 2.3 |
| Yes, medical appointments/medications | 1.4 | -5.9, 8.7 | 3.21 | 1.03, 10.7 | -0.20 | -1.9, 1.5 | 1.1 | -0.32, 2.5 |
| Yes, non-medical meetings/appointments | 2.8 | -2.8, 8.4 | 1.30 | 0.56, 3.05 | -0.04 | -1.3, 1.2 | 0.64 | -0.42, 1.7 |
| **In the last month, did you put off or neglect going somewhere** **due to distance or lack of transportation?** |  |  |  |  |  |  |  |  |
| No | 0.00 | — | 1.00 | — | 0.00 | — | 0.00 | — |
| Yes | 1.6 | -2.8, 6.0 | 1.02 | 0.53, 1.98 | -0.21 | -1.2, 0.79 | 0.62 | -0.22, 1.5 |
| **In the past 12 months, has the electric, gas, oil, or** **water company threatened to shut off services in your home?** |  |  |  |  |  |  |  |  |
| No | 0.00 | — | 1.00 | — | 0.00 | — | 0.00 | — |
| *Yes, or already shut off** | *-2.1* | *-6.6, 2.4* | *1.56* | *0.79, 3.10* | *0.57* | *-0.45, 1.6* | *0.19* | *-0.68, 1.1* |
| Yes | -3.1 | -7.7, 1.5 | 1.48 | 0.73, 3.03 | 0.61 | -0.45, 1.7 | 0.00 | -0.89, 0.88 |
| Already shut off | 6.9 | -4.3, 18 | 2.30 | 0.45, 13.0 | 0.24 | -2.3, 2.8 | 1.9 | -0.32, 4.0 |
| **In the last month, how hard was it for you to pay for the** **very basics like food, housing, medical care, and heating?** |  |  |  |  |  |  |  |  |
| Not hard | 0.00 | — | 1.00 | — | 0.00 | — | 0.00 | — |
| *Hard** | *1.1* | *-4.0, 6.1* | *0.6* | *0.28, 1.28* | *0.43* | *-0.71, 1.6* | *0.89* | *-0.07, 1.8* |
| Somewhat hard | 0.83 | -4.4, 6.1 | 0.55 | 0.24, 1.21 | 0.81 | -0.36, 2.0 | 0.78 | -0.22, 1.8 |
| Very hard | 1.7 | -4.9, 8.3 | 0.76 | 0.28, 2.05 | -0.63 | -2.1, 0.84 | 1.2 | -0.08, 2.4 |
| **How often do you feel isolated from others?** |  |  |  |  |  |  |  |  |
| No (Hardly ever) | 0.00 | — | 1.00 | — | 0.00 | — | 0.00 | — |
| *Yes** | *0.69* | *-3.9, 5.3* | *1.09* | *0.55, 2.18* | *0.11* | *-0.93, 1.1* | *0.32* | *-0.56, 1.2* |
| Some of the time | -0.75 | -5.8, 4.3 | 1.26 | 0.58, 2.73 | 0.16 | -1.0, 1.3 | 0.12 | -0.86, 1.1 |
| Often | 2.8 | -2.8, 8.4 | 0.89 | 0.38, 2.06 | 0.04 | -1.2, 1.3 | 0.61 | -0.47, 1.7 |
| **How often do you see or talk to people that you care about and feel close to?** |  |  |  |  |  |  |  |  |
| 5 or more times a week | 0.00 | — | 1.00 | — | 0.00 | — | 0.00 | — |
| 3 to 5 times a week | -4.0 | -9.6, 1.7 | 0.74 | 0.32, 1.71 | 0.31 | -0.97, 1.6 | -0.43 | -1.5, 0.66 |
| 1 or 2 times a week | -1.8 | -7.3, 3.7 | 0.74 | 0.32, 1.67 | -0.56 | -1.8, 0.70 | -0.16 | -1.2, 0.91 |
| *At least once a week** | *2.4* | *-4.6, 9.3* | *1.04* | *0.35, 3.03* | *0.17* | *-1.4, 1.8* | *-0.47* | *-1.8, 0.87* |
| Less than once a week | -4.1 | -12, 3.5 | 0.80 | 0.25, 2.54 | -0.25 | -2.0, 1.5 | 0.29 | -1.2, 1.8 |
| Abbreviations: CI = Confidence Interval, OR = Odds Ratio  * = Binarized comparison of means and confidence intervals between the reference point and all other possible characteristics per measure | | | | | | | | |
