# Supplementary material for: Evaluating user experiences with an AI chatbot for health-related social needs: A cross-sectional mixed methods study
Source: PLOS Digit Health. 2026 Oct 30;5(10):e0001789. doi: 10.1371/journal.pdig.0001789 (PMC13722182; doi:10.1371/journal.pdig.0001789)
Supplement: Supplementary file 5 [file pdig.0001789.s005.docx]

# **S5 Appendix.** Frequency of user behaviors

## Table A. Frequency of assistive buttons before leaving a chat session

| **Assistive Button** | **N** |
| --- | --- |
| Coverage | 34 |
| Find Help for Something Else | 31 |
| More Resources | 30 |
| Cost | 26 |
| Business Hours | 20 |
| Availability | 16 |
| Change My Location | 2 |

## Table B. Patterns in resource-seeking behaviors with specificity

| **Category** | **Specific Resource** | **Purpose for the Resource** | **Personal Background** | **Multiple Needs** | **Thoughts on Finding a Resource** |
| --- | --- | --- | --- | --- | --- |
| Mean  (SD) | 0.37  (0.48) | 0.02  (0.13) | 0.03  (0.16) | 0.07  (0.26) | 0.91  (0.28) |
| Median | 0 | 0 | 0 | 0 | 1 |
| IQR | 1 | 0 | 0 | 0 | 0 |

## Table C. Frequencies of keywords in free text messages

| **Word** | **N** |
| --- | --- |
| program(s) | 217 |
| shelter(s) | 118 |
| residential | 108 |
| food | 80 |
| nutrition(al) | 76 |
| looking | 47 |
| related | 38 |
| pantry(ies) | 37 |
| health | 35 |
| resource(s) | 35 |

## Table D. Topics of interest

| **Topic** | **N** |
| --- | --- |
| money | 31 |
| housing | 30 |
| food | 28 |
| work | 14 |
| transit | 10 |
| education | 9 |
| goods | 7 |
| health | 6 |
| legal | 3 |
| care | 2 |
| *(other unique topics)*  *utilities, recreational activities, marketplaces, delivery services, dealerships, job market* | *30* |
