# Supplementary material for: Evaluating user experiences with an AI chatbot for health-related social needs: A cross-sectional mixed methods study
Source: PLOS Digit Health. 2026 Oct 30;5(10):e0001789. doi: 10.1371/journal.pdig.0001789 (PMC13722182; doi:10.1371/journal.pdig.0001789)
Supplement: Supplementary file 6 [file pdig.0001789.s006.docx]

# **S6 Appendix.** Qualitative domains and themes

| **Domain** | **Theme** | **Quote** | **Participant#** |
| --- | --- | --- | --- |
| Knowledge | Use of links & follow-up | “I can use the phone numbers or links to follow up later on this resource.” | 24 |
|  | Scheduling & eligibility | “I want to make sure the resource I choose is open when I can get there.” | 95 |
|  | Location preferences | “If it's too far, then I want to adjust the range like other apps do.” | 106 |
| Delivery | Interface usability | “The interface and language were very intuitive and simple.” | 45 |
|  | Response speed | “It was speedy and quick in response to my many questions.” | 37 |
|  | Voice interaction | “Maybe it could break it down for me by communicating via voice.” | 18 |
|  | Resource fit evaluation | “It kinda sorted out my needs for me.” | 91 |
| Positive Feedback | Ease of use | “The chatbot was easy to use.” | 73 |
|  | Resource richness | “I loved how I was provided with more than 2 services.” | 115 |
|  | Guidance | “I like how it gave suggestions on info to look up.” | 9 |
| Negative Feedback | Relevance issues | “Some of the resources listed were not very helpful for me and other family members.” | 126 |
|  | Voice absence | “The only negative I have is that the chatbot couldn’t ‘talk’ to me aloud.” | 56 |
|  | Location mismatch | “The resource results included places that wasn’t in my area of interest.” | 22 |
| Suggestions | Clarify search process | “It should help me reduce my queries to a question rather than many.” | 90 |
|  | Age-targeted suggestions | “It could provide options that are better suited for young adults.” | 67 |
|  | Refine prompts | “It should start by helping me be more specific when typing a question.” | 3 |
