# Supplementary material for: Evaluating user experiences with an AI chatbot for health-related social needs: A cross-sectional mixed methods study
Source: PLOS Digit Health. 2026 Oct 30;5(10):e0001789. doi: 10.1371/journal.pdig.0001789 (PMC13722182; doi:10.1371/journal.pdig.0001789)
Supplement: Supplementary file 7 [file pdig.0001789.s007.docx]

# **S7 Appendix.** Chatbot helpfulness and desired future features

| **Resource Helpfulness** | **N** | **%** |
| --- | --- | --- |
| Yes, helpful for me or someone else. | 120 | 94% |
| No, unhelpful for me or someone else. | 8 | 6% |
| **Desired App Features** | |  |
| History & Favorites Tab | 87 | 19% |
| Profile Creation | 80 | 17% |
| Password Protected App | 70 | 15% |
| Daily Tips & Personalized Updates | 69 | 15% |
| Multilingual Support | 57 | 12% |
| Connections to Your Medical Records | 50 | 11% |
| Phone Notifications | 48 | 10% |
| Other Features | 4 | 1% |
| Words of Encouragement | 1 |  |
| Chatbot Personalities | 1 |  |
| Technical Guidance | 2 |  |
| Total Votes | 465 | 100% |
