# Supplementary material for: Evaluating user experiences with an AI chatbot for health-related social needs: A cross-sectional mixed methods study
Source: PLOS Digit Health. 2026 Oct 30;5(10):e0001789. doi: 10.1371/journal.pdig.0001789 (PMC13722182; doi:10.1371/journal.pdig.0001789)
Supplement: Supplementary file 8 [file pdig.0001789.s008.docx]

# **S8 Appendix.** Forest plots of demographics, usability, and health-related social needs

**Demographic Factors and User Experience**

In terms of relationships which are not monotonically scaling but suggest a general shift, having income >=$16k correlates with increased Trust (Mean class of 2.97-3.12 compared to the mean class of 2.90 for incomes <$10k). Likewise, having any post-secondary degree trends negatively for usability relative to the RV (Range of difference in estimated means = -3.72 to -5.91). We additionally see that compared to the RV (No Children), usability (Range of difference in estimated means = 6.58 to 16.00) trust (Mean class of 3.08 to 3.44 compared to 2.49 for no children), and satisfaction (Range of difference in estimated means = 0.14 to 2.25) trend positively with having any children while task load (Range of difference in estimated means = -1.33 to -1.7) correlates negatively.

**Health-related Social Needs and User Experience**

In terms of notable non-monotonic shifts, a decrease in quantity of food eaten the household (RV: Enough food that we want to eat) trended with increased trust (Mean class estimate ranging from 2.95 to 3.30 compared to the mean class of 2.96 for ‘Enough of the kinds of food we want to eat’). Reports of utility shutoff trend positively with task load (Estimated difference from 0.24 to 0.61 compared to no risk of shutoff), and satisfaction (Estimated difference from 0 to 1.86 compared to no risk of shutoff). Lastly, increase in interactions with family or friends positively with usability (Estimated difference from 0.13 to 4.11 compared to less than once a week) and trust (estimated mean class for 3.15 for 5+ times a week compared to 3.06 for less than once a week).

## Fig A. Demographics and Usability Forest Plot

## Fig B. Demographics and Trust Forest Plot

## Fig C. Demographics and Task Load Forest Plot

## Fig D. Demographics and Satisfaction Forest Plot

## Fig E. Health-related Social Need and Usability Forest Plot

## Fig F. Health-related Social Need and Trust Forest Plot

## Fig G. Health-related Social Need and Task Load Forest Plot

## Fig H. Health-related Social Need and Satisfaction Forest Plot
