# Supplementary material for: Evaluating user experiences with an AI chatbot for health-related social needs: A cross-sectional mixed methods study
Source: PLOS Digit Health. 2026 Oct 30;5(10):e0001789. doi: 10.1371/journal.pdig.0001789 (PMC13722182; doi:10.1371/journal.pdig.0001789)
Supplement: Supplementary file 9 [file pdig.0001789.s009.docx]

# **S9 Appendix.** Pairwise comparisons between demographics, usability, and health-related social needs

## Table A. Education and Usability Pairwise Comparison

| **contrast** | **estimate** | **SE** | **lower.CL** | **upper.CL** |
| --- | --- | --- | --- | --- |
| Some college, no degree - Highschool | 1.02 | 3.06 | -7.85 | 9.89 |
| Associate degree - Highschool | -3.72 | 3.55 | -14.00 | 6.56 |
| Associate degree - Some college, no degree | -4.74 | 3.27 | -14.19 | 4.72 |
| Bachelors degree - Highschool | -5.91 | 3.40 | -15.74 | 3.93 |
| Bachelors degree - Some college, no degree | -6.92 | 3.10 | -15.90 | 2.05 |
| Bachelors degree - Associate degree | -2.19 | 3.58 | -12.56 | 8.18 |
| Masters degree - Highschool | -4.89 | 4.76 | -18.66 | 8.89 |
| Masters degree - Some college, no degree | -5.91 | 4.55 | -19.08 | 7.27 |
| Masters degree - Associate degree | -1.17 | 4.89 | -15.33 | 12.99 |
| Masters degree - Bachelors degree | 1.02 | 4.78 | -12.82 | 14.86 |

## Table B. Income and Usability Pairwise Comparison

| **contrast** | **estimate** | **SE** | **lower.CL** | **upper.CL** |
| --- | --- | --- | --- | --- |
| ($10,000-$15,999) - Less than $10,000 | -8.46 | 7.75 | -29.91 | 12.99 |
| ($16,000-$19,999) - Less than $10,000 | -4.12 | 4.60 | -16.85 | 8.61 |
| ($16,000-$19,999) - ($10,000-$15,999) | 4.33 | 8.05 | -17.95 | 26.62 |
| ($20,000-$29,999) - Less than $10,000 | -2.24 | 3.47 | -11.86 | 7.38 |
| ($20,000-$29,999) - ($10,000-$15,999) | 6.22 | 7.46 | -14.45 | 26.88 |
| ($20,000-$29,999) - ($16,000-$19,999) | 1.88 | 4.10 | -9.48 | 13.25 |
| ($30,000-$39,999) - Less than $10,000 | 0.40 | 3.33 | -8.81 | 9.60 |
| ($30,000-$39,999) - ($10,000-$15,999) | 8.85 | 7.40 | -11.63 | 29.33 |
| ($30,000-$39,999) - ($16,000-$19,999) | 4.52 | 3.98 | -6.50 | 15.54 |
| ($30,000-$39,999) - ($20,000-$29,999) | 2.64 | 2.60 | -4.57 | 9.84 |

## Table C. Children and Usability Pairwise Comparison

| **contrast** | **estimate** | **SE** | **lower.CL** | **upper.CL** |
| --- | --- | --- | --- | --- |
| (1-2) - None | 6.58 | 6.35 | -9.96 | 23.12 |
| (3-4) - None | 7.45 | 6.77 | -10.17 | 25.08 |
| (3-4) - (1-2) | 0.87 | 2.93 | -6.76 | 8.51 |
| 5 or more - None | 16.00 | 10.78 | -12.08 | 44.08 |
| 5 or more - (1-2) | 9.42 | 8.89 | -13.74 | 32.58 |
| 5 or more - (3-4) | 8.55 | 9.20 | -15.40 | 32.50 |

## Table D. Education and Task Load Pairwise Comparison

| **contrast** | **estimate** | **SE** | **lower.CL** | **upper.CL** |
| --- | --- | --- | --- | --- |
| Some college, no degree - Highschool | -1.27 | 0.68 | -3.22 | 0.69 |
| Associate degree - Highschool | -0.32 | 0.78 | -2.59 | 1.95 |
| Associate degree - Some college, no degree | 0.94 | 0.72 | -1.15 | 3.03 |
| Bachelors degree - Highschool | 0.89 | 0.75 | -1.28 | 3.06 |
| Bachelors degree - Some college, no degree | 2.15 | 0.68 | 0.17 | 4.13 |
| Bachelors degree - Associate degree | 1.21 | 0.79 | -1.08 | 3.50 |
| Masters degree - Highschool | 1.91 | 1.05 | -1.13 | 4.95 |
| Masters degree - Some college, no degree | 3.17 | 1.00 | 0.27 | 6.08 |
| Masters degree - Associate degree | 2.23 | 1.08 | -0.89 | 5.36 |
| Masters degree - Bachelors degree | 1.02 | 1.05 | -2.04 | 4.07 |

## Table E. Income and Task Load Pairwise Comparison

| **contrast** | **estimate** | **SE** | **lower.CL** | **upper.CL** |
| --- | --- | --- | --- | --- |
| ($10,000-$15,999) - Less than $10,000 | -0.36 | 1.77 | -5.25 | 4.54 |
| ($16,000-$19,999) - Less than $10,000 | 0.74 | 1.05 | -2.17 | 3.64 |
| ($16,000-$19,999) - ($10,000-$15,999) | 1.10 | 1.84 | -3.99 | 6.18 |
| ($20,000-$29,999) - Less than $10,000 | 0.97 | 0.79 | -1.23 | 3.16 |
| ($20,000-$29,999) - ($10,000-$15,999) | 1.33 | 1.70 | -3.39 | 6.04 |
| ($20,000-$29,999) - ($16,000-$19,999) | 0.23 | 0.94 | -2.37 | 2.82 |
| ($30,000-$39,999) - Less than $10,000 | 0.92 | 0.76 | -1.18 | 3.03 |
| ($30,000-$39,999) - ($10,000-$15,999) | 1.28 | 1.69 | -3.39 | 5.96 |
| ($30,000-$39,999) - ($16,000-$19,999) | 0.19 | 0.91 | -2.33 | 2.70 |
| ($30,000-$39,999) - ($20,000-$29,999) | -0.04 | 0.59 | -1.68 | 1.60 |

## Table F. Children and Task Load Pairwise Comparison

| **contrast** | **estimate** | **SE** | **lower.CL** | **upper.CL** |
| --- | --- | --- | --- | --- |
| (1-2) - None | -1.38 | 1.45 | -5.16 | 2.40 |
| (3-4) - None | -1.70 | 1.55 | -5.73 | 2.33 |
| (3-4) - (1-2) | -0.32 | 0.67 | -2.07 | 1.42 |
| 5 or more - None | -1.33 | 2.46 | -7.75 | 5.08 |
| 5 or more - (1-2) | 0.04 | 2.03 | -5.25 | 5.34 |
| 5 or more - (3-4) | 0.36 | 2.10 | -5.11 | 5.84 |

## Table G. Education and Satisfaction Pairwise Comparison

| **contrast** | **estimate** | **SE** | **lower.CL** | **upper.CL** |
| --- | --- | --- | --- | --- |
| Some college, no degree - Highschool | 0.67 | 0.60 | -1.06 | 2.40 |
| Associate degree - Highschool | -0.30 | 0.69 | -2.30 | 1.71 |
| Associate degree - Some college, no degree | -0.97 | 0.64 | -2.82 | 0.88 |
| Bachelors degree - Highschool | -0.31 | 0.66 | -2.23 | 1.61 |
| Bachelors degree - Some college, no degree | -0.98 | 0.60 | -2.74 | 0.77 |
| Bachelors degree - Associate degree | -0.01 | 0.70 | -2.04 | 2.01 |
| Masters degree - Highschool | -0.11 | 0.93 | -2.80 | 2.58 |
| Masters degree - Some college, no degree | -0.78 | 0.89 | -3.35 | 1.79 |
| Masters degree - Associate degree | 0.19 | 0.95 | -2.58 | 2.95 |
| Masters degree - Bachelors degree | 0.20 | 0.93 | -2.50 | 2.90 |

## Table H. Income and Satisfaction Pairwise Comparison

| **contrast** | **estimate** | **SE** | **lower.CL** | **upper.CL** |
| --- | --- | --- | --- | --- |
| ($10,000-$15,999) - Less than $10,000 | -3.05 | 1.47 | -7.13 | 1.02 |
| ($16,000-$19,999) - Less than $10,000 | -0.22 | 0.87 | -2.64 | 2.20 |
| ($16,000-$19,999) - ($10,000-$15,999) | 2.83 | 1.53 | -1.40 | 7.07 |
| ($20,000-$29,999) - Less than $10,000 | -0.30 | 0.66 | -2.13 | 1.53 |
| ($20,000-$29,999) - ($10,000-$15,999) | 2.75 | 1.42 | -1.18 | 6.68 |
| ($20,000-$29,999) - ($16,000-$19,999) | -0.08 | 0.78 | -2.24 | 2.08 |
| ($30,000-$39,999) - Less than $10,000 | 0.34 | 0.63 | -1.41 | 2.09 |
| ($30,000-$39,999) - ($10,000-$15,999) | 3.39 | 1.41 | -0.50 | 7.28 |
| ($30,000-$39,999) - ($16,000-$19,999) | 0.56 | 0.76 | -1.54 | 2.65 |
| ($30,000-$39,999) - ($20,000-$29,999) | 0.64 | 0.49 | -0.73 | 2.01 |

## Table I. Children and Satisfaction Pairwise Comparison

| **contrast** | **estimate** | **SE** | **lower.CL** | **upper.CL** |
| --- | --- | --- | --- | --- |
| (1-2) - None | 0.14 | 1.23 | -3.05 | 3.33 |
| (3-4) - None | 0.66 | 1.31 | -2.74 | 4.06 |
| (3-4) - (1-2) | 0.52 | 0.57 | -0.95 | 1.99 |
| 5 or more - None | 2.25 | 2.08 | -3.17 | 7.67 |
| 5 or more - (1-2) | 2.11 | 1.72 | -2.36 | 6.58 |
| 5 or more - (3-4) | 1.59 | 1.77 | -3.03 | 6.21 |

## Table J. Food Quantity and Usability Pairwise Comparison

| **contrast** | **estimate** | **SE** | **lower.CL** | **upper.CL** |
| --- | --- | --- | --- | --- |
| Enough but not always the kinds of food we want - Enough of the kinds of food we want to eat | 0.59 | 2.65 | -6.33 | 7.50 |
| Sometimes not enough to eat - Enough of the kinds of food we want to eat | 1.98 | 3.74 | -7.75 | 11.71 |
| Sometimes not enough to eat - Enough but not always the kinds of food we want | 1.39 | 3.34 | -7.29 | 10.08 |
| Often not enough to eat - Enough of the kinds of food we want to eat | 9.59 | 5.18 | -3.90 | 23.09 |
| Often not enough to eat - Enough but not always the kinds of food we want | 9.01 | 4.90 | -3.76 | 21.77 |
| Often not enough to eat - Sometimes not enough to eat | 7.61 | 5.56 | -6.87 | 22.10 |

## Table K. Utility Shutoff and Usability Pairwise Comparison

| **contrast** | **estimate** | **SE** | **lower.CL** | **upper.CL** |
| --- | --- | --- | --- | --- |
| Yes - No | -3.10 | 2.32 | -8.61 | 2.40 |
| Already shut off - No | 6.93 | 5.69 | -6.56 | 20.42 |
| Already shut off - Yes | 10.04 | 5.82 | -3.77 | 23.84 |

## Table L. Needs Affordability and Usability Pairwise Comparison

| **contrast** | **estimate** | **SE** | **lower.CL** | **upper.CL** |
| --- | --- | --- | --- | --- |
| Somewhat hard - Not hard at all | 0.83 | 2.66 | -5.49 | 7.15 |
| Very hard - Not hard at all | 1.72 | 3.34 | -6.19 | 9.64 |
| Very hard - Somewhat hard | 0.90 | 2.91 | -6.00 | 7.79 |

## Table M. Social Isolation and Usability Pairwise Comparison

| **contrast** | **estimate** | **SE** | **lower.CL** | **upper.CL** |
| --- | --- | --- | --- | --- |
| 5 or more times a week - 3 to 5 times a week | 3.98 | 2.84 | -3.43 | 11.38 |
| 5 or more times a week - 1 or 2 times a week | 1.79 | 2.80 | -5.49 | 9.08 |
| 5 or more times a week - Less than once a week | 4.11 | 3.82 | -5.84 | 14.06 |
| 3 to 5 times a week - 1 or 2 times a week | -2.18 | 2.98 | -9.94 | 5.57 |
| 3 to 5 times a week - Less than once a week | 0.13 | 3.95 | -10.16 | 10.43 |
| 1 or 2 times a week - Less than once a week | 2.32 | 3.92 | -7.89 | 12.53 |

## Table N. Food Quantity and Task Load Pairwise Comparison

| **contrast** | **estimate** | **SE** | **lower.CL** | **upper.CL** |
| --- | --- | --- | --- | --- |
| Enough but not always the kinds of food we want - Enough of the kinds of food we want to eat | 0.22 | 0.60 | -1.35 | 1.78 |
| Sometimes not enough to eat - Enough of the kinds of food we want to eat | -0.87 | 0.85 | -3.07 | 1.34 |
| Sometimes not enough to eat - Enough but not always the kinds of food we want | -1.08 | 0.76 | -3.05 | 0.89 |
| Often not enough to eat - Enough of the kinds of food we want to eat | -1.75 | 1.17 | -4.81 | 1.31 |
| Often not enough to eat - Enough but not always the kinds of food we want | -1.96 | 1.11 | -4.86 | 0.93 |
| Often not enough to eat - Sometimes not enough to eat | -0.88 | 1.26 | -4.16 | 2.40 |

## Table O. Utility Shutoff and Task Load Pairwise Comparison

| **contrast** | **estimate** | **SE** | **lower.CL** | **upper.CL** |
| --- | --- | --- | --- | --- |
| Yes - No | 0.61 | 0.53 | -0.66 | 1.87 |
| Already shut off - No | 0.24 | 1.31 | -2.86 | 3.34 |
| Already shut off - Yes | -0.37 | 1.34 | -3.54 | 2.81 |

## Table P. Needs Affordability and Task Load Pairwise Comparison

| **contrast** | **estimate** | **SE** | **lower.CL** | **upper.CL** |
| --- | --- | --- | --- | --- |
| Somewhat hard - Not hard at all | 0.81 | 0.59 | -0.60 | 2.22 |
| Very hard - Not hard at all | -0.63 | 0.74 | -2.40 | 1.13 |
| Very hard - Somewhat hard | -1.44 | 0.65 | -2.98 | 0.09 |

## Table Q. Social Isolation and Task Load Pairwise Comparison

| **contrast** | **estimate** | **SE** | **lower.CL** | **upper.CL** |
| --- | --- | --- | --- | --- |
| 5 or more times a week - 3 to 5 times a week | -0.31 | 0.65 | -2.00 | 1.38 |
| 5 or more times a week - 1 or 2 times a week | 0.56 | 0.64 | -1.10 | 2.23 |
| 5 or more times a week - Less than once a week | 0.25 | 0.87 | -2.02 | 2.52 |
| 3 to 5 times a week - 1 or 2 times a week | 0.87 | 0.68 | -0.90 | 2.64 |
| 3 to 5 times a week - Less than once a week | 0.56 | 0.90 | -1.79 | 2.91 |
| 1 or 2 times a week - Less than once a week | -0.31 | 0.89 | -2.64 | 2.02 |

## Table R. Food Quantity and Satisfaction Pairwise Comparison

| **contrast** | **estimate** | **SE** | **lower.CL** | **upper.CL** |
| --- | --- | --- | --- | --- |
| Enough but not always the kinds of food we want - Enough of the kinds of food we want to eat | 0.06 | 0.51 | -1.28 | 1.39 |
| Sometimes not enough to eat - Enough of the kinds of food we want to eat | 0.66 | 0.72 | -1.22 | 2.55 |
| Sometimes not enough to eat - Enough but not always the kinds of food we want | 0.61 | 0.64 | -1.07 | 2.29 |
| Often not enough to eat - Enough of the kinds of food we want to eat | 1.48 | 1.00 | -1.13 | 4.09 |
| Often not enough to eat - Enough but not always the kinds of food we want | 1.42 | 0.95 | -1.04 | 3.89 |
| Often not enough to eat - Sometimes not enough to eat | 0.82 | 1.08 | -1.99 | 3.62 |

## Table S. Utility Shutoff and Satisfaction Pairwise Comparison

| **contrast** | **estimate** | **SE** | **lower.CL** | **upper.CL** |
| --- | --- | --- | --- | --- |
| Yes - No | -0.00 | 0.45 | -1.07 | 1.06 |
| Already shut off - No | 1.86 | 1.10 | -0.75 | 4.47 |
| Already shut off - Yes | 1.87 | 1.13 | -0.80 | 4.54 |

## Table T. Needs Affordability and Satisfaction Pairwise Comparison

| **contrast** | **estimate** | **SE** | **lower.CL** | **upper.CL** |
| --- | --- | --- | --- | --- |
| Somewhat hard - Not hard at all | 0.78 | 0.51 | -0.42 | 1.98 |
| Very hard - Not hard at all | 1.18 | 0.63 | -0.33 | 2.68 |
| Very hard - Somewhat hard | 0.39 | 0.55 | -0.92 | 1.71 |

## Table U. Social Isolation and Satisfaction Pairwise Comparison

| **contrast** | **estimate** | **SE** | **lower.CL** | **upper.CL** |
| --- | --- | --- | --- | --- |
| 5 or more times a week - 3 to 5 times a week | 0.43 | 0.55 | -1.01 | 1.87 |
| 5 or more times a week - 1 or 2 times a week | 0.16 | 0.54 | -1.25 | 1.58 |
| 5 or more times a week - Less than once a week | -0.29 | 0.74 | -2.22 | 1.64 |
| 3 to 5 times a week - 1 or 2 times a week | -0.27 | 0.58 | -1.77 | 1.24 |
| 3 to 5 times a week - Less than once a week | -0.72 | 0.77 | -2.72 | 1.27 |
| 1 or 2 times a week - Less than once a week | -0.46 | 0.76 | -2.44 | 1.52 |
