# Supplementary material for: Evaluating user experiences with an AI chatbot for health-related social needs: A cross-sectional mixed methods study
Source: PLOS Digit Health. 2026 Oct 30;5(10):e0001789. doi: 10.1371/journal.pdig.0001789 (PMC13722182; doi:10.1371/journal.pdig.0001789)
Supplement: Supplementary file 10 [file pdig.0001789.s010.docx]

# **S10 Appendix.** Class estimates of demographics, usability, and health-related social needs

## Table A. Education Mean Trust Class Estimate

| **education** | **mean.class** | **SE** | **asymp.LCL** | **asymp.UCL** |
| --- | --- | --- | --- | --- |
| Highschool | 2.99 | 0.16 | 2.68 | 3.29 |
| Some college, no degree | 3.24 | 0.11 | 3.02 | 3.46 |
| Associate degree | 3.01 | 0.16 | 2.70 | 3.32 |
| Bachelors degree | 2.94 | 0.16 | 2.63 | 3.25 |
| Masters degree | 3.06 | 0.27 | 2.54 | 3.58 |
| None of the above | 2.04 | 0.70 | 0.68 | 3.41 |

## Table B. Income Mean Trust Class Estimate

| **income** | **mean.class** | **SE** | **asymp.LCL** | **asymp.UCL** |
| --- | --- | --- | --- | --- |
| Less than $10,000 | 2.90 | 0.19 | 2.53 | 3.26 |
| $10,000-$15,999 | 2.02 | 0.48 | 1.08 | 2.95 |
| $16,000-$19,999 | 2.97 | 0.22 | 2.54 | 3.41 |
| $20,000-$29,999 | 3.19 | 0.12 | 2.95 | 3.42 |
| $30,000-$39,999 | 3.12 | 0.10 | 2.93 | 3.32 |

## Table C. Kids Mean Trust Class Estimate

| **kids** | **mean.class** | **SE** | **asymp.LCL** | **asymp.UCL** |
| --- | --- | --- | --- | --- |
| None | 2.49 | 0.38 | 1.73 | 3.24 |
| 1-2 | 3.08 | 0.08 | 2.92 | 3.23 |
| 3-4 | 3.14 | 0.17 | 2.82 | 3.46 |
| 5 or more | 3.44 | 0.45 | 2.56 | 4.32 |

## Table D. Food Quantity Mean Trust Class Estimate

| **contrast** | **estimate** | **SE** | **lower.CL** | **upper.CL** |
| --- | --- | --- | --- | --- |
| Enough but not always the kinds of food we want - Enough of the kinds of food we want to eat | 0.22 | 0.60 | -1.35 | 1.78 |
| Sometimes not enough to eat - Enough of the kinds of food we want to eat | -0.87 | 0.85 | -3.07 | 1.34 |
| Sometimes not enough to eat - Enough but not always the kinds of food we want | -1.08 | 0.76 | -3.05 | 0.89 |
| Often not enough to eat - Enough of the kinds of food we want to eat | -1.75 | 1.17 | -4.81 | 1.31 |
| Often not enough to eat - Enough but not always the kinds of food we want | -1.96 | 1.11 | -4.86 | 0.93 |
| Often not enough to eat - Sometimes not enough to eat | -0.88 | 1.26 | -4.16 | 2.40 |

## Table E. Utility Shutoff Mean Trust Class Estimate

| **contrast** | **estimate** | **SE** | **lower.CL** | **upper.CL** |
| --- | --- | --- | --- | --- |
| Yes - No | 0.61 | 0.53 | -0.66 | 1.87 |
| Already shut off - No | 0.24 | 1.31 | -2.86 | 3.34 |
| Already shut off - Yes | -0.37 | 1.34 | -3.54 | 2.81 |

## Table F. Needs Affordability Mean Trust Class Estimate

| **contrast** | **estimate** | **SE** | **lower.CL** | **upper.CL** |
| --- | --- | --- | --- | --- |
| Somewhat hard - Not hard at all | 0.81 | 0.59 | -0.60 | 2.22 |
| Very hard - Not hard at all | -0.63 | 0.74 | -2.40 | 1.13 |
| Very hard - Somewhat hard | -1.44 | 0.65 | -2.98 | 0.09 |

## Table G. Social Isolation Mean Trust Class Estimate

| **contrast** | **estimate** | **SE** | **lower.CL** | **upper.CL** |
| --- | --- | --- | --- | --- |
| 5 or more times a week - 3 to 5 times a week | -0.31 | 0.65 | -2.00 | 1.38 |
| 5 or more times a week - 1 or 2 times a week | 0.56 | 0.64 | -1.10 | 2.23 |
| 5 or more times a week - Less than once a week | 0.25 | 0.87 | -2.02 | 2.52 |
| 3 to 5 times a week - 1 or 2 times a week | 0.87 | 0.68 | -0.90 | 2.64 |
| 3 to 5 times a week - Less than once a week | 0.56 | 0.90 | -1.79 | 2.91 |
| 1 or 2 times a week - Less than once a week | -0.31 | 0.89 | -2.64 | 2.02 |
