# Supplementary material for: Evaluating user experiences with an AI chatbot for health-related social needs: A cross-sectional mixed methods study
Source: PLOS Digit Health. 2026 Oct 30;5(10):e0001789. doi: 10.1371/journal.pdig.0001789 (PMC13722182; doi:10.1371/journal.pdig.0001789)
Supplement: Supplementary file 11 [file pdig.0001789.s011.docx]

# **S11 Appendix.** Large language model system prompts and parameters

| **Chat Progress** | **LLM** | **Prompt** |
| --- | --- | --- |
| Describing Search Strategy | Claude 3.5 Haiku (temp = 0.1, token cap = 53) | In first person, tell the user that you will look for resources related to {search variable} in {location variable}  End the message with “Here’s what I found:” |
| Define the Search Category | GPT-3.5 Turbo (temp = 0.7, token cap = 128) | The user has provided a statement that may or may not contain specifics about the resource they are looking for.  Here is the statement: {last utterance variable}  Here is the list of resources:  food: community gardens, emergency food, food delivery, food pantry, meals, help pay for food, government food benefits, nutrition education  housing: temporary shelter, weather relief, help find housing, help pay for housing, help pay for utilities, help pay for internet or phone, home & renters insurance, housing vouchers, maintenance & repairs, efficiency upgrades, pest control, housing advice, foreclosure counseling, homebuyer education, residential housing, long-term housing, assisted living, independent living, nursing home, public housing, safe housing, short-term housing, sober living  goods: baby supplies, baby clothes, diapers & formula, clothing, clothes for school, clothes for work, clothing vouchers, home goods, blankets & fans, books, furniture, home fuels, efficient appliances, personal care items, supplies for school, supplies for work, medical supplies, assistive technology, prosthesis, personal safety, toys & gifts  transit: help pay for transit, bus passes, help pay for car, help pay for gas, transportation, transportation for healthcare, transportation for school  health: addiction & recovery (12-step, detox, drug testing, medications for addiction, outpatient treatment, peer recovery coaching, residential treatment, sober living, substance abuse counseling), dental care, end-of-life care (bereavement, burial & funeral help, hospice), health education, disease management, family planning, nutrition education, parenting education, safety education, sex education, understand disability, understand mental health, help pay for healthcare, disability benefits, discounted healthcare, health insurance, prescription assistance, transportation for healthcare, medical care (alternative medicine, checkup & test, disability screening, disease screening, hearing tests, pregnancy tests), exercise & fitness, vaccinations, primary care, skilled nursing, support & service animals, mental health care (anger management, counseling, group therapy, family counseling, individual counseling, medications, mental health evaluation, outpatient treatment, psychiatric emergency services, residential treatment, support animals)  sexual & reproductive health: family planning, birth control, fertility, sex education, std/sti treatment & prevention, hiv treatment, women's health, maternity care, postnatal care vision care  money: financial assistance, help pay for childcare, help pay for food, government food benefits, help pay for healthcare, disability benefits, discounted healthcare, prescription assistance, help pay for housing, help pay for utilities, help pay for internet or phone, home & renters insurance, help pay for school (books, clothes, supplies, transportation), help pay for transit, help pay for gas, help pay for work expenses, government benefits (disability, food, retirement, unemployment), financial education (credit counseling, foreclosure counseling, homebuyer education, savings program), insurance (health, home & renters), loans (business, personal), tax preparation  care: adoption & foster care (placement, parenting, planning, counseling, post-adoption support), animal welfare, community support services, computer or internet access, exercise & fitness, recreation, daytime care (adult daycare, after school care, before school care, childcare), help pay for childcare, early childhood intervention, head start, preschool, relief for caregivers, end-of-life care (bereavement, burial & funeral help, hospice), navigating the system (help fill out forms, help find housing, case management), physical safety (disaster response, emergency food, temporary shelter, weather relief, help find missing persons, immediate safety, help escape violence, safe housing, safety education), residential care (assisted living, residential treatment, nursing home), overnight camp, support network (counseling, help hotlines, home visiting, in-home support, mentoring, one-on-one support, peer recovery coaching, peer support, spiritual support, support groups, virtual support)  education: help find school, help pay for school (books, clothes, financial aid & loans, supplies, transportation), more education (alternative education, civic engagement, college readiness, disaster preparedness & response, english as a second language, environmental education, financial education, foreign languages, ged/high-school equivalency, health education, special education, tutoring, youth development), citizenship & immigration, skills & training (basic literacy, computer class, daily life skills, interview training, resume development, skills assessment, specialized training), supported employment  work: help find work, job placement, supported employment, help pay for work expenses (clothes, supplies), unemployment benefits, skills & training (basic literacy, computer class, ged/high-school equivalency, interview training, resume development, skills assessment, specialized training), workplace rights  legal: advocacy & legal aid, adoption & foster care (placement, parenting, planning, counseling, post-adoption support), citizenship & immigration, discrimination & civil rights, guardianship, identification recovery, government programs, workplace rights, mediation, notary, representation, translation & interpretation.  Match the statement with one of the resources listed try to be as specific as possible with the resource. Output only the resource and omit any additional text or instructions. If no specific resources are specified output the character "0" and do not break that rule.  Examples:  statement: resources for finding work  output: help find work  statement: I need some help with resources to fix my teeth  output: dental care  statement: I'm not sure what I want  output: 0  statement: I want to find a coffee house near me  output: 0 |
| Clean Search Results | GPT-3.5 Turbo (temp = 0, token cap = 25) | List the names of 3 services separated by comas.  Input Variables:  {1^st^ result variable}, {2^nd^ result variable}, {3^rd^ result variable} |
| Describe provided resources (Continue) | Claude 3.5 Haiku (temp = 1, token cap = 200) | Using the descriptions of each service ({1st result variable}, {2nd result variable}, {3rd result variable}) the user will begin to explore their options. Reply with context of {need} or transition.  Buttons will appear to redirect or specify further requests.  More Details: read {resource topic} and draft a reply with the context of {user need variable}  Contact Info: read {resource topic} and draft a reply containing {resource contact}. Include a recommendation for next best window of opportunity to access resources.  Open Maps: Sends within the chat an embedded path via Google Maps or Apple Maps depending on device compatibility. Do not reply.  Change the User’s Zip Code: a script will change the conversation state, do not reply.  Other Resources in My Location: describe extra results not included in the original search from a previous Clean Search Results output found in {all results}  If {state} is “finale” then reply with the following and ask for justification based on any negative reply:  “Were you able to find the information you were looking for today? |
| Describe provided resources  (Finish Chat) | GPT-3.5 Turbo (temp 0.2, token cap = 25) | Using the descriptions of each service ({1st result variable}, {2nd result variable}, {3rd result variable}) the user will begin to explore their options. Reply with context of {need} or transition.  Buttons will appear to redirect or specify further requests.  More Details: Sets {topic} to corresponding {full description}  Contact Info: Sets {topic} to corresponding {contact info}  Open Maps: Sends within the chat an embedded path via Google Maps or Apple Maps depending on device compatibility. Reply with embedded script found in {map preview}  Change the User’s Zip Code: set {state} to “zipcode” and transition with {zip} script  Other Resources in My Location: remove previously described resources from {all results}  If the user expresses satisfaction or a need to exit the session, set {state} to “finale” |
